# Lifestyle behaviors and risk of cardiovascular disease and prognosis among individuals with cardiovascular disease: a systematic review and meta-analysis of 71 prospective cohort studies

**DOI:** 10.1186/s12966-024-01586-7

**Published:** 2024-04-22

**Authors:** Jian Wu, Yifei Feng, Yuanyuan Zhao, Zhiping Guo, Rongmei Liu, Xin Zeng, Fan Yang, Bei Liu, Jianqing Gu, Clifford Silver Tarimo, Weihao Shao, Xinghong Guo, Quanman Li, Lipei Zhao, Mingze Ma, Zhanlei Shen, Qiuping Zhao, Yudong Miao

**Affiliations:** 1 Department of Health management, College of Public Health, Zhengzhou University, Zhengzhou, Henan People’s Republic of China; 2 Central China Fuwai Hospital of Zhengzhou University, Zhengzhou, Henan People’s Republic of China; 3 School of Public Health, NHC Key Lab of Health Technology Assessment, Fudan University, Shanghai, People’s Republic of China; 4 Department of Laboratorial Science and Technology & Vaccine Research Center, School of Public Health, Peking University, Beijing, People’s Republic of China; 5 Healthy Lifestyle Medicine Research Center, School of Medicine, Southern University of Science and Technology, Shenzhen, Guangdong People’s Republic of China; 6 Department of Science and Laboratory Technology, Dar es Salaam Institute of Technology, P.O. Box 2958, Dar es Salaam, Tanzania

**Keywords:** Lifestyle behaviors, Cardiovascular disease, Meta-analysis

## Abstract

**Background:**

Healthy lifestyle behaviors (LBs) have been widely recommended for the prevention and management of cardiovascular disease (CVD). Despite a large number of studies exploring the association between combined LBs and CVD, a notable gap exists in integration of relevant literatures. We conducted a systematic review and meta-analysis of prospective cohort studies to analyze the correlation between combined LBs and the occurrence of CVD, as well as to estimate the risk of various health complications in individuals already diagnosed with CVD.

**Methods:**

Articles published up to February 10, 2023 were sourced through PubMed, EMBASE and Web of Science. Eligible prospective cohort studies that reported the relations of combined LBs with pre-determined outcomes were included. Summary relative risks (RRs) and 95% confidence intervals (CIs) were estimated using either a fixed or random-effects model. Subgroup analysis, meta-regression, publication bias, and sensitivity analysis were as well performed.

**Results:**

In the general population, individuals with the healthiest combination of LBs exhibited a significant risk reduction of 58% for CVD and 55% for CVD mortality. For individuals diagnosed with CVD, adherence to the healthiest combination of LBs corresponded to a significant risk reduction of 62% for CVD recurrence and 67% for all-cause mortality, when compared to those with the least-healthy combination of LBs. In the analysis of dose-response relationship, for each increment of 1 healthy LB, there was a corresponding decrease in risk of 17% for CVD and 19% for CVD mortality within the general population. Similarly, among individuals diagnosed with CVD, each additional healthy LB was associated with a risk reduction of 27% for CVD recurrence and 27% for all-cause mortality.

**Conclusions:**

Adopting healthy LBs is associated with substantial risk reduction in CVD, CVD mortality, and adverse outcomes among individuals diagnosed with CVD. Rather than focusing solely on individual healthy LB, it is advisable to advocate for the adoption of multiple LBs for the prevention and management of CVD.

**Trial registration:**

PROSPERO: CRD42023431731.

**Supplementary Information:**

The online version contains supplementary material available at 10.1186/s12966-024-01586-7.

## Significance

The meta-analysis examined the quantitative correlation between lifestyle behaviors (LBs) and the incidence of cardiovascular disease (CVD) and CVD mortality in the general population, as well as adverse outcomes in CVD patients. The results suggest that healthy LBs are associated with substantial risk reduction in these 3 outcomes. Multiple LBs, instead of tackling one certain LB, should be recommended for the prevention and management of CVD.

**Supplementary Information:**

The online version contains supplementary material available at 10.1186/s12966-024-01586-7.

## Background

Cardiovascular disease (CVD) has been one of the major global health concerns for decades [1, 2]. In 2017, it was estimated that approximately 1.76 billion people worldwide were affected by CVD [3]. Currently, both high-income and low-and middle-income countries are witnessing an increase in disease burden associated with CVD morbidity and mortality [4]. The World Health Organization (WHO) reported that the global annual deaths caused by CVD are approximately 17.9 million, accounting for 32% of all deaths [1]. The treatment and management of CVD may be costly, limiting the health and sustainable development of every country in the world [5]. CVD causes annual global economic losses of at least one trillion dollars [6]. To date, cost-effective interventions and health policies are imperative to reduce premature mortality and treatment costs caused by CVD.

CVD is largely recognized as a preventable disease, due to the facts that modifiable risk factors have been shown to account for more than 90% of the risk of CVD [7]. Globally, an increasing number of healthy lifestyle behaviors (LBs) have been proven to be effective in preventing and even treating CVD. Cohort studies have shown that LBs such as maintaining a healthy diet, engaging in regular physical activity, maintaining a healthy body weight, avoiding tobacco use, getting quality sleep, and fostering social interactions, are cost-effective strategies for modifying risk factors of CVD including dyslipidemia, high blood pressure, and elevated glucose levels [8–12]. The significant effects of healthy LBs in managing CVD are being increasingly confirmed and reiterated in numerous literature sources. The symptoms of CVD have been reported to improve and lessen following regular interventions comprising dietary adjustments and physical exercise [13, 14]. Moreover, some prospective cohort studies are uncovering the benefits of the number of LBs in lowering the incidence, mortality, and long-term adverse outcomes of CVD [12, 15–19].

However, to the best of our knowledge, there is currently no comprehensive meta-analysis quantifying the dose-response relationships between LBs and incident CVD and risk of health outcomes among individuals with CVD. In this study, we gathered the prospective cohort studies of healthy LBs for prevention and treatment of CVD published worldwide since 1998. We hence conducted a meta-analysis to evaluate the quantitative correlation between LBs and the incidence of CVD and CVD mortality in the general population, as well as adverse outcomes in individuals with CVD. The current study is expected to provide higher-level evidence supporting the positive role of healthy LBs in reducing the risk of CVD and promoting favorable clinical treatment outcomes for individuals with CVD.

## Methods

The current systematic review and meta-analysis was conducted and reported in accordance with the Preferred Reporting Items for Systematic Reviews and Meta-Analyses (PRISMA) guidelines (See Additional file 1) [20]. In drafting the abstract, we adhered to the 12-item PRISMA (Preferred Reporting Items for Systematic Reviews and Meta-Analyses) extension guidelines [21]. This meta-analysis was registered with the International Prospective Register of Systematic Reviews (PROSPERO) under the registration number CRD42023431731.

### Data source and search strategy

A comprehensive search was conducted on PubMed, EMBASE, and Web of Science databases up until February 10, 2023, to identify relevant studies reporting on the association between LBs and the incidence of total or subtypes of CVD. The search also aimed to explore the risk of total or subtypes of CVD mortality, total or subtypes of CVD recurrence, CVD mortality, or all-cause mortality among individuals with CVD. The search was limited to studies published in the English language, using a combination of MeSH terms and free-text terms (See Supplemental Table 1 in Additional file 2). We manually combed through the reference lists of the included articles to identify additional pertinent research. Published systematic reviews and meta-analyses were also used as a data source. Two investigators (JW and YF) independently conducted systematic searches, screened the articles, and reviewed the full text of the selected articles. In case of any disagreement, they discussed the discrepancies with the senior investigator to reach a consensus (YM).

### Study selection

Studies were included in this meta-analysis if they met the following inclusion criteria: (1) prospective cohort study design; (2) adult population including the general population or individuals with CVD; (3) studies with a minimum follow-up duration of more than 1 year; (4) studies focusing on healthy lifestyle with three or more LBs, including those derived from the American Heart Association’s Life’s Essential 8 framework [22], excluding metabolic factors, such as blood lipid and glucose; (5) each LB in the studies was the categorical variable and different categories was assigned unequal value; (6) the studies reporting pre-determined outcomes, including incident of total CVD or CVD subtypes (including coronary heart disease [CHD], stroke, heart failure [HF], ischemic heart disease [IHD] or myocardial infarction [MI]), total or subtypes of CVD mortality in general population, CVD recurrence, CVD mortality, or all-cause mortality among individuals with CVD; (7) the studies reported quantitative estimates (odds ratio [OR], risk ratio [RR], or hazard ratio [HR]) and their 95% confidence intervals (CIs), or provided sufficient data to calculate these estimates. In cases where multiple publications were based on the same dataset, those with more complete information were selected. Otherwise, publications that included the largest number of participants were selected. Moreover, reviews, comments, letters, and editorials were excluded from the analysis. Additionally, we excluded reviews, comments, letters, and editorials.

### Data extraction and quality assessment

Data were extracted from published articles with the use of a predefined protocol. Two investigators (JW and YF) independently extracted the following information from the included studies: first author, publication year, country, cohort name, sex, mean age, duration of follow-up, sample size, the definitions of combination of LBs, number of cases, outcome attainment, health status, number of cases and person-years/number of participants per LB category, most adjusted risk estimates (ORs, RRs, or HRs) with their corresponding 95% CIs for each category and adjustment variables. Any disagreement was resolved by consensus involving a third author. For articles with insufficient data or unclear information, the corresponding authors were contacted (at least two attempts were made).

The study quality of eligible prospective cohort acticles was evaluated using the Newcastle-Ottawa Scale [23], with a total score of 9 points (highest quality) for eight aspects, which focused on the selection of the study groups (4 points), the comparability of the groups (2 points) and the ascertainment of outcome (3 points).

### Definitions of LB

LB refers to health-related lifestyle behaviors [24]. Our study defines combined LBs as consisting of three or more LBs, such as smoking, drinking alcohol or drinking moderately, sleep, physical exercise, diet, body weight, etc. Importantly, all LBs are regarded as equally significant in their contribution to overall health outcomes. A comprehensive LB score was obtained by assigning values to each LB. Due to the varying number of categorical divisions within each LB across the eligible studies, there are two main scoring methodologies in our meta-analysis: (1) studies simply classify individuals either exhibiting or not exhibiting a certain behavior as “1” or “0”. This method exists in the studies of dividing each LB into two categories. (2) studies assign unequal value to a certain behavior with different categories, in cases where featured LB into multiple categories. An example of this methodology is evident in the segmentation of physical activity into five categories, ranging from “rarely or never” to “4 or more times per week”, with corresponding scores ranging from 0 to 4 assigned for each category. Due to we were unable to access the original data contained within the articles, we could not differentiate the varying degree of effect of different LBs on the outcome, treating all LBs as having equal significance in their contribution. Similarly, we did not prespecify cutoffs for each LB, instead relying on the definitions provided by the respective study authors. We considered the largest number of healthy LBs in the original study as “the healthiest combination of LBs”, and similarly considered the least number of healthy LBs as “the least-healthy combination of LBs”.

### Data synthesis and analysis

Relative risks (RRs) were used as the unified effect measure to assess the association between the LBs and the pre-determined outcomes. In some studies, hazard ratios (HRs) were reported and were considered approximately equal to RRs in terms of measuring the association [25, 26]. Due to the high incidence of CVD, the ORs may present an overestimation of the true RRs; therefore, we converted the ORs reported by included studies into RRs using a previously published correction method [27]. Articles reporting data separately from different cohorts, or from different regions, or reporting different types of outcomes within an article, were treated as separate studies. For articles reported data separately for different subgroups such as different sex or sub-types of outcomes, the fixed-effect model was used to re-calculate risk estimates. In cases where the number of cases or participants in each category was not explicitly provided, we calculated it using the available data [28]. When the category with the least-healthy combination of LBs was not the reference category, the method of Hamling and colleagues was used to re-calculate the risk estimates [29]. When exposures were reported as a range, we took the midpoint value for analyses. In situations where the healthiest combination of LBs and the least-healthy combination of LBs categories were open-ended, we followed a specific approach. For the least-healthy combination of LBs category, we defined 0 as the lower bound, while for the healthiest combination of LBs category, we used the number of LBs involved in the study as the upper bound. We then estimated the midpoint value accordingly to assigned values to these categories for the purpose of analysis [30].

We first used random-effects models to estimate the pooled RRs and 95%CIs for the healthiest versus the least-healthy combination of LBs and CVD incidence, mortality in general population, and CVD recurrence, CVD mortality, or all-cause mortality among individuals with CVD. We calculated study-specific slopes (linear trends) and 95% CIs from the natural logs of the reported RRs and CIs across categories of combination of LBs by using the method of Greenland [31] and the random-effects model to pool the study-specific dose-response effect estimates [32]. Study-specific effect estimates were calculated per 1 healthy LB increament. Only studies with at least three levels of combination of LBs and one point assigned to each healthy LB of the binary categories were included in the dose-response analysis.

Heterogeneity was tested by Cochran Q and *I*^2^ statistics [33]. A *p* < 0.10 was considered statistically significant for the Q statistic. By using a cut-off of 0.10, the issue of the Q statistic being less effective in detecting true heterogeneity was addressed, and it also helped to reduce the risk of committing a type II error [34]. *I*^2^ values of approximately 25%, 50%, and 75% were considered to reflect low, moderate, and high heterogeneity, respectively. Prespecified subgroup analyses and meta-regression by sub-types of outcomes, continent, sex, follow-up year, average age, factors included in LB score (smoking, alcohol drinking, physical activity, diet, and body weight), adjustments for age, economic level, and educational level were performed to access potential sources of heterogeneity. We also performed the sensitivity analyses by removing one study at a time to evaluate the robustness of the summary estimate. Egger’s test and funnel plot were both used to detect any evidence of publication bias for each meta-analysis [35]. In case significant publication bias was detected, we used the trim and fill method to make the adjustments [36]. Subgroup analyses, sensitivity analyses, and publication bias assessments were not conducted if there were fewer than 8 cohort comparisons available.

All analyses were performed using Stata 14.0 (Stata Corp, College Station, TX, USA). All tests were two-sided, with *P* < 0.05 considered statistically significant.

## Results

### Literature search and study characteristics

**Fig. 1:**
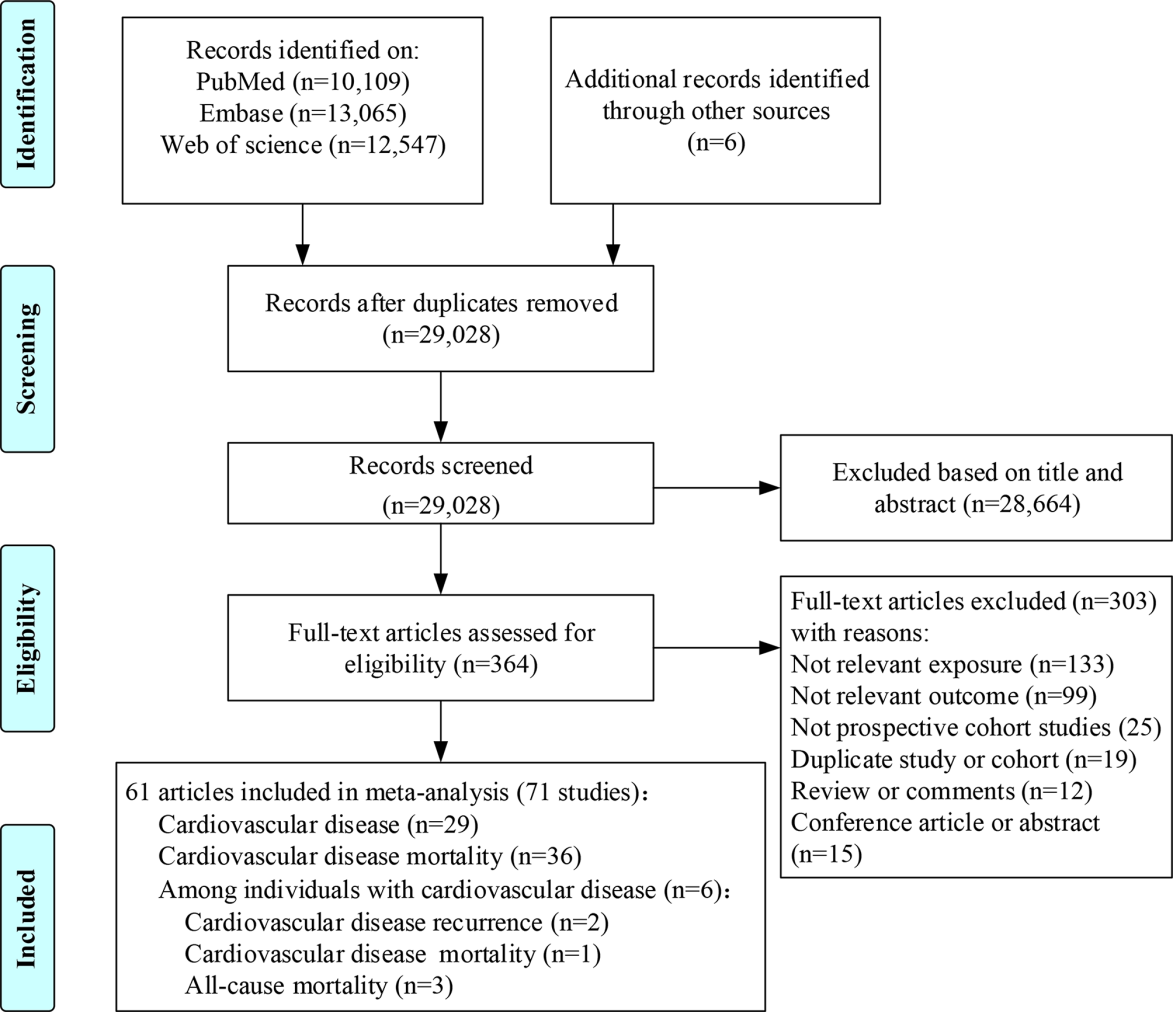
Flowchart of article selection for the meta-analysis

Literature search processes are summarized in Fig. 1. We identified 35,727 potential eligible articles. After removing duplicate articles (*n* = 6,699) and conducting titles or abstracts screening (*n* = 29,028), 364 articles were retrieved for critical full-text review. Finally, an overview of 61 articles (a total of 71 studies: 29 on CVD incidence, 36 on CVD mortality in general population; 2 on CVD recurrence, 1 CVD mortality, and 3 all-cause mortality among individuals with CVD) was included in the meta-analysis, representing a total of 6,163,255 participants with the average age ranged from 26.5 to 72 years. Among these articles, 2 articles reporting data from various cohorts [37, 38], 2 reporting different regions [39, 40], and 6 reporting different outcomes [16, 18, 19, 41–43], were treated as independent studies. The sample size of the cohorts ranged from 388 to 903,499, and the duration of follow-up ranged from 2.4 to 37 years. Geographically, 22 studies were conducted in Asia [15, 17, 19, 40, 44–60], 25 in Europe [8, 9, 16, 38, 40, 43, 61–75], and 24 in the United States [11, 37, 39, 41, 76–87]. 24 studies combined 5 or more main LBs [11, 18, 19, 37, 44–46, 48, 54, 58, 59, 61, 63–65, 68, 72, 76–78, 82, 85, 86, 88], and 60 studies reported that combinations of LBs had at least 3 levels and each LB was assigned a score, which meet the inclusion criteria for dose–response analyses [8, 9, 11, 15–19, 37–52, 55–60, 62, 63, 65–68, 70, 71, 73–75, 78–81, 86–89]. Table 1 shows the main characteristics of the included studies and Supplemental Tables 2–4 in Additional file 2 show the definition and categories of LBs. The mean (range) quality score was 7.07, assessed using the Newcastle-Ottawa Scale for cohort studies (See Supplemental Table 5 in Additional file 2).

**Table 1:** Characteristics of included studies

Author (year)	Country/region	Mean/median follow-up, years	Sex	Mean age, years	Sample size	No. of outcomes	Combination of LBs					
							Smoking	Alcohol drinking	PA	Diet	Body weight	Others
**Studies on CVD incidence and mortality**												
Mo, M.(2023)	Sweden	12	M/F	26.5	15,411	CVD 222	✓	✓	✓	✓	✓	
Mao, Ziling (2023)	US	11	M/F	63.8	15,467	CVD 1,563	✓	✓	✓	✓	✓	✓
Zuo, Y. (2022)	China	11.03	M/F	51.6	94,831	CVD 6,590	✓	✓	✓	✓		✓
Heath, L. (2022)	UK	11	M/F	56.2	339,913	CVD 29,545 CVD mortality 4,655	✓	✓	✓	✓		
Guasch-Ferré, Marta (2022 NHS)	US	30	F	45.4	67,250	CVD 6,984	✓	✓	✓	✓	✓	✓
Guasch-Ferré, Marta (2022 HPFS)	US	30	M	56.2	29,114	CVD 4,726	✓	✓	✓	✓	✓	✓
Yang, R. (2021)	China	10	M/F	51.5	487,197	HF 4,208	✓	✓	✓	✓	✓	
Han, Y. (2021)	China	11.2	M/F	51.2	461,047	IHD 34,304 Stroke 37,727	✓	✓	✓	✓	✓	
Tsai, Ming-Chieh (2021)	Taiwan, China	14.3	M/F	43	6,042	CVD 520	✓	✓	✓	✓	✓	
Dimovski, K. (2019)	Sweden	18	M	57.88	26,333	CHD 3,417	✓		✓	✓	✓	
Diaz-Gutierrez, J. (2018)	Spain	10.4	M/F	37.32	19,336	CVD 140	✓	✓	✓	✓	✓	✓
Lv, J. (2017)	China	7.2	M/F	50.7	461,211	CHD 3,331 Stroke 19,348 IHD 21,857	✓	✓	✓	✓	✓	
Larsson, S. C. (2016 CSM)	Sweden	13	M	59.3	33,966	HF 1,488	✓		✓	✓	✓	
Larsson, S. C. (2016 SMC)	Sweden	13	F	60.9	30,713	HF 1,096	✓		✓	✓	✓	
Chomistek, A. K. (2015)	US	20	M/F	37.1	88,940	CHD 456	✓	✓	✓	✓	✓	✓
Del Gobbo, L. C. (2015)	US	21.5	M/F	72	4,490	HF 1,380	✓	✓	✓	✓	✓	
Akesson, A. (2014)	Sweden	11	M	58	20,721	MI 1,361	✓	✓	✓	✓	✓	
Agha, G. (2014)	US	11	M/F	63.54	84,537	HF 1,826	✓		✓	✓	✓	
Larsson, S. C. (2014)	Sweden	10.4	F	60.95	31,696	Stroke 1,554	✓	✓	✓	✓	✓	
Carlsson, A.C. (2013)	Sweden	10.85	M/F	60	3,741	CVD 375	✓	✓	✓	✓		
Ahmed, H. M. (2013)	US	7.6	M/F	64	6,229	CHD 358	✓		✓	✓	✓	
Hoevenaar-Blom, M. P. (2013)	Netherlands	12	M/F	42	14,639	CVD 607 CVD mortality 129	✓	✓	✓	✓		✓
Wang, Y. (2011)	Finland	14.1	M/F	46.31	38,075	HF 1,083	✓		✓	✓	✓	
Zhang, Y. (2011)	Finland	13.7	M/F	45.81	36,686	Stroke 1,478	✓	✓	✓	✓	✓	
Ford, E. S. (2009)	Germany	7.8	M/F	49.3	23,153	Stroke 195 MI 214	✓		✓	✓	✓	
Cardi, M. (2009)	US	37	M/F	61.27	963	CVD 249	✓	✓	✓		✓	✓
Lee, C. D. (2009)	US	30	M	44.13	23,657	CHD 482 CVD mortality 306	✓		✓		✓	
Myint, P. K. (2009)	UK	11.5	M/F	58.27	20,040	Stroke 599	✓	✓	✓	✓		
Kurth, T. (2006)	US	10	F	54.6	37,636	Stroke 450	✓	✓	✓	✓	✓	
Troeschel, A. N. (2023)	US	10.3	M/F	64.4	18,484	CVD mortality 1,216	✓	✓	✓		✓	
Wang, T. (2022)	China	6	M/F	59.1	11,247	CVD mortality 375	✓	✓	✓	✓		
Hu, P. (2022)	China	4	M/F	58.36	11,395	CVD mortality 64	✓	✓	✓	✓	✓	✓
Kim, S. (2022)	Korea	9.6	F	70.6	3,034	CVD mortality 137	✓	✓	✓		✓	
Ibsen, D. B. (2021)	Denmark	17	M/F	56.1	54,276	CVD mortality 1,753	✓	✓	✓	✓		
Li, Z. (2021)	US	22	F	61.36	33,155	CVD mortality 6,574	✓	✓	✓		✓	
Liu, G. (2021)	China	10	M/F	87.5	15,349	CVD mortality 1,010	✓	✓	✓	✓		
Sotos-Prieto, M. (2021)	Spain	8.7	M/F	46.45	11,090	CVD mortality 74	✓	✓	✓	✓	✓	✓
Troeschel, A. N. (2021)	US	10.3	M/F	64.2	17,465	CVD mortality 1,170	✓	✓	✓	✓	✓	✓
Zhang, X. (2021)	China	2.4	M/F	55.9	903,499	CVD mortality 3,474	✓	✓	✓	✓		
Lee, D. H. (2020)	Korea	6.01	M/F	50.62	37,472	CVD mortality 213	✓	✓	✓		✓	✓
Wu, M. Y. (2020)	China	12.13	M/F	56.4	331,457	CVD mortality 3,143	✓	✓	✓		✓	
Bonaccio, M. (2019)	Italy	8.2	M/F	47.7	22,839	CVD mortality 444	✓		✓	✓	✓	
Zhu, N. (2019)	China	10.2	M/F	51.5	487,198	IHD mortality 5,116 Stroke mortality 6,081	✓	✓	✓	✓	✓	
Han, C. (2018)	China	7.24	M/F	51.64	93,987	CVD mortality 1,383	✓		✓	✓	✓	
Li, Y. (2018)	US	27.2	M/F	48.96	123,219	CVD mortality 10,689	✓	✓	✓	✓	✓	
Zhang, Q. L. (2017)	China	9.29	M	55.34	59,747	CVD mortality 1,637	✓	✓	✓	✓		
Fazel-Tabar Malekshah, A. (2016)	Iran	8.08	M/F	51.54	40,708	CVD mortality 1,407			✓	✓	✓	
Lohse, T. (2016)	Switzerland	21.7	M/F	46.05	13,159	CVD mortality 828		✓	✓	✓	✓	✓
Warren Andersen, S. (2016 AF)	US	7	M/F	51	79,101	CVD mortality 1,462	✓	✓	✓			✓
Warren Andersen, S. (2016 WH)	US	7	M/F	51	79,101	CVD mortality 587	✓	✓	✓			✓
Taheri, Shahrad (2015 EU)	Europe	21	M/F	53.05	1,065	CVD mortality 243	✓	✓	✓	✓		
Taheri, Shahrad (2015 SA)	South Asia	21	M/F	50.78	970	CVD mortality 328	✓	✓	✓	✓		
Hoevenaar-Blom, M. P. (2014)	Netherlands	12	M/F	42	171,866	CVD mortality 129	✓	✓	✓	✓		✓
Eguchi, E. (2012)	Japan	16.5	M/F	56.1	43,010	CVD mortality 1,907 CHD mortality 402 Stroke mortality 849	✓	✓	✓	✓	✓	✓
Ford, E. S. (2011)	US	14	M/F	46.75	15,416	CVD mortality 1,182	✓	✓	✓	✓		
McCullough, M. L. (2011)	US	14	M/F	63.6	52,670	CVD mortality 5,628		✓	✓	✓	✓	
Odegaard, A. O. (2011)	Singapore	20.6	M/F	55.3	44,056	CVD mortality 1,971	✓	✓	✓	✓	✓	✓
Kvaavik, E. (2010)	England, Wales, and Scotland	20	M/F	43.7	4,886	CVD mortality 431	✓	✓	✓	✓		
Mitchell, J. A. (2010)	US	16.1	M	43.8	38,110	CVD mortality 949	✓	✓	✓		✓	✓
Nechuta, S. J. (2010)	China	9.1	F	52.19	71,243	CVD mortality 605	✓		✓	✓	✓	
Lee, C. D. (2009)	US	30	M	44.13	23,657	CVD mortality 306	✓		✓		✓	
Khaw, K. T. (2008)	UK	11	M/F	58.13	20,244	CVD mortality 676	✓	✓	✓	✓		
Knoops, K. T. (2004)	Europe	10	M/F	74.24	2,339	CVD mortality 371	✓	✓	✓	✓		
Luoto, R. (1998)	Finland	15	M/F	39.5	18,974	CVD mortality 1,005	✓		✓	✓		
**Studies on CVD recurrence, mortality and all-cause mortality among individuals with CVD**												
Yang, Y. L. (2021)	Taiwan, China	2.23	M/F	66.5	716	CVD recurrence 175	✓		✓	✓	✓	
Booth, J. N. (2014)	US	4.3	M/F	68.77	4,174	CHD recurrence 448 All-cause mortality 746	✓		✓	✓	✓	
Towfighi, A. (2012)	US	6	M/F	67	388	CVD mortality 126 All-cause mortality 208	✓	✓	✓	✓	✓	
Han, Y. (2021)	China	11.2	M/F	51.2	461,047	All-cause mortality 12,597	✓	✓	✓	✓	✓	

*Abbreviations* CVD, cardiovascular disease; LB, lifestyle behaviors; PA, physical activity; US, United States; M, male; F, female; HF, heart failure; IHD, ischemic heart disease; CHD, coronary heart disease; MI, myocardial infarction; UK, United Kingdom; NHS, Nurses’ Health Study; HPFS, Health Professionals Follow-up Study; AF, African American; WH, White; CSM, Cohort of Swedish Men; SMC, Swedish Mammography Cohort

### Association of LBs with incident CVD in general population

**Fig. 2:**
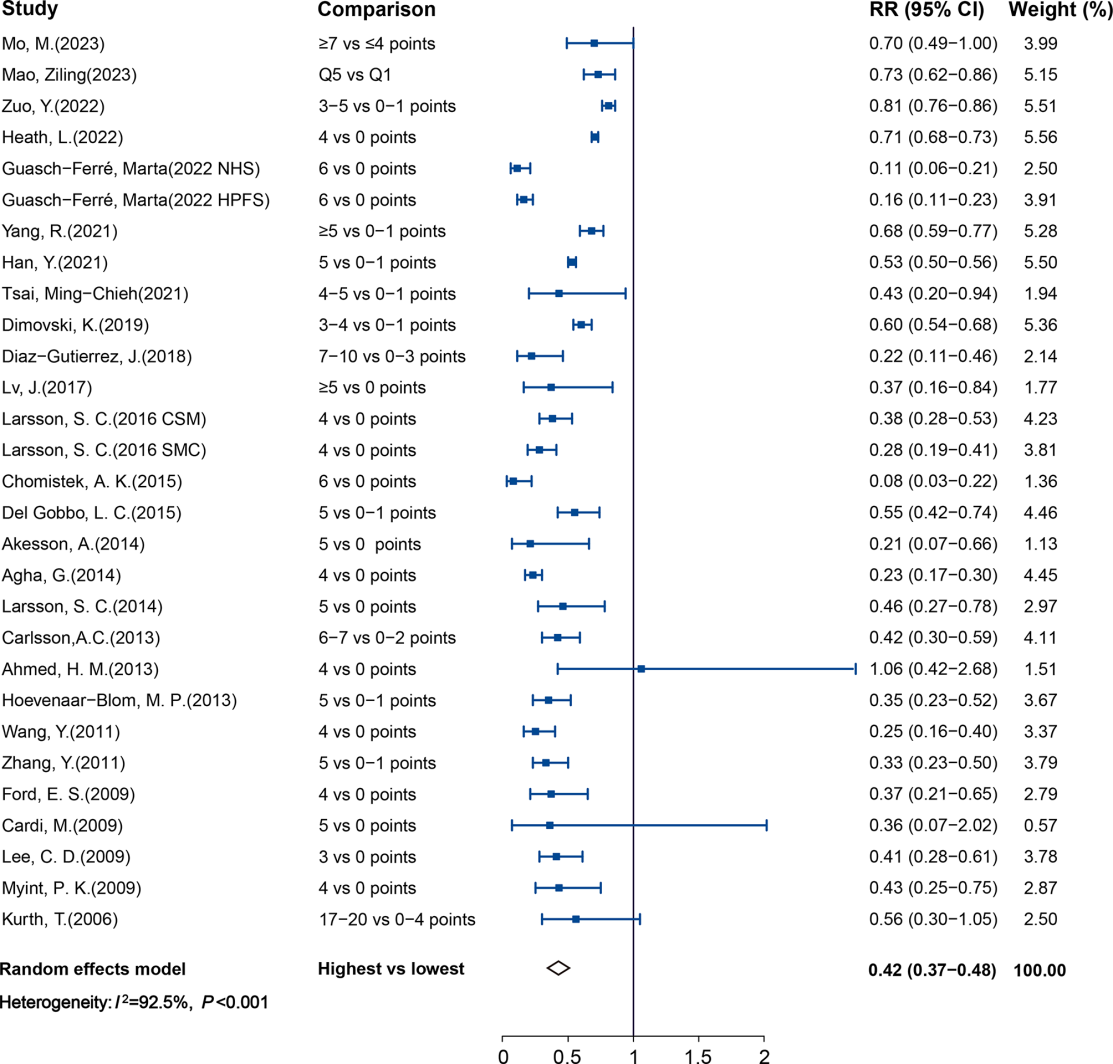
Forest plot of pooled relative risk for CVD with the healthiest versus the least-healthy combination of LBs

**Fig. 3:**
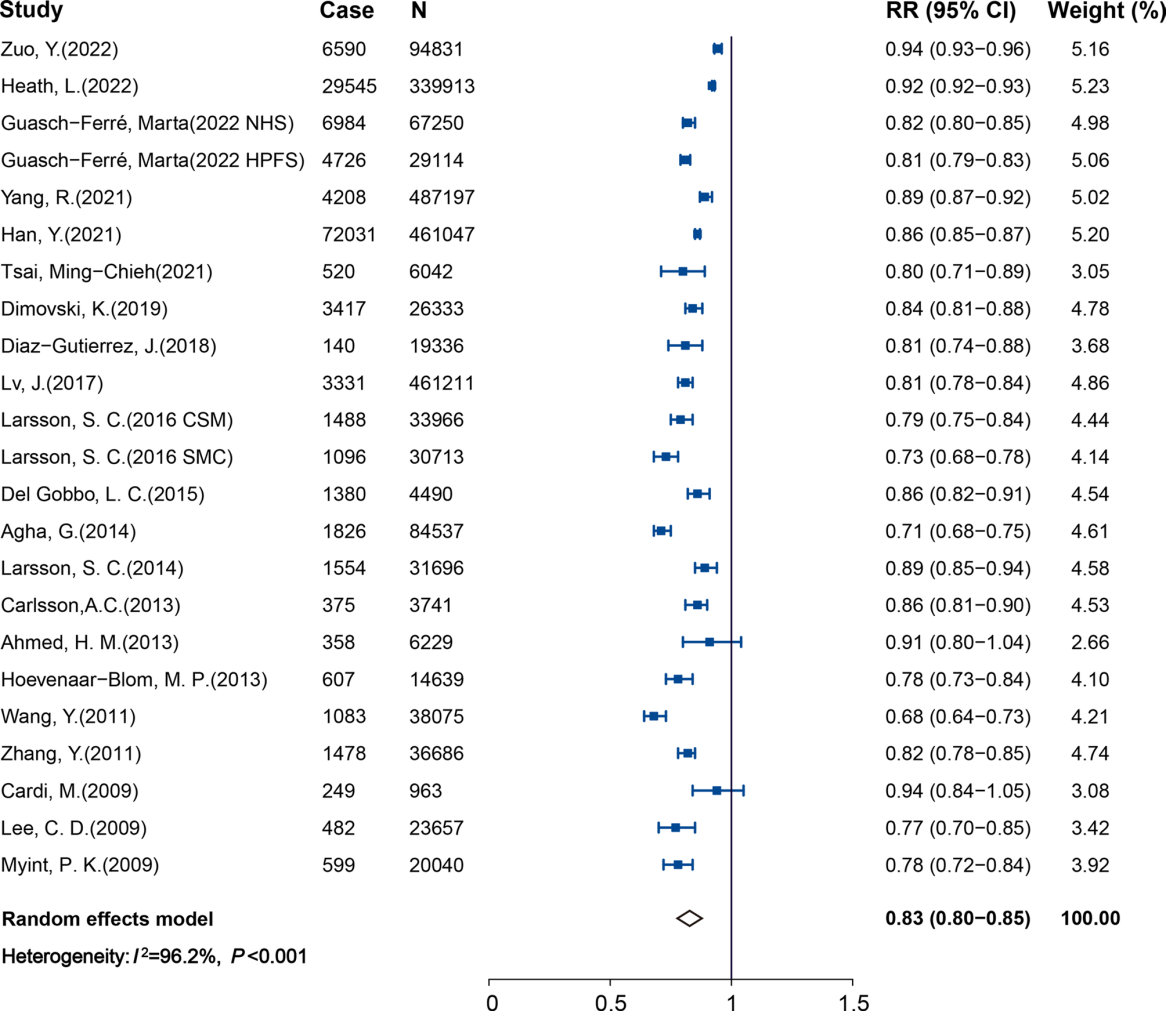
Forest plot for the pooled association between per 1 healthy LB increment and CVD

Twenty-nine studies (2,523,034 participants and 189,733 cases) reported results comparing participants with the healthiest vs. least-healthy combination of LBs. The pooled RR and 95% CI was 0.42 (0.37–0.48), with high heterogeneity found (*I*^2^ = 92.5%, *P*_heterogeneity_ < 0.001; Fig. 2). Publication bias was detected using Egger’s test (*P* < 0.05). The trim-and-fill method was then conducted to adjust for the asymmetry, which weakened the protection effect but left the direction unchanged (RR: 0.62; 95% CI: 0.55–0.71; See Supplemental Fig. 1A in Additional file 2). Data from twenty-three studies (2,321,706 participants and 144,067 cases) were included in the dose-response analysis of LBs and CVD. The pooled RR was 0.83 (95% CI, 0.80–0.85) with per 1 healthy LB increment, with significant heterogeneity (*I*^2^ = 96.2%, *P*_heterogeneity_ < 0.001; Fig. 3). We detected statistically significant publication bias by Egger’s test (*P* < 0.05), with application of the trim and fill method, the protection effect did not change (RR: 0.90; 95% CI: 0.87–0.92; See Supplemental Fig. 1B in Additional file 2).

Considering the high heterogeneity across the included studies, we carried out meta-regression and subgroup analyses to explore the potential sources of heterogeneity. Meta-regression indicating that sex, adjustment for economic level and educational level may explain the high heterogeneity across studies in the healthiest vs. least-healthy combination of LBs (all *P*_regression_<0.05; See Supplemental Table 6 in Additional file 2), and factors included in LB score with or without alcohol drinking (*P*_regression_ = 0.005) may the source of heterogeneity in the dose-response relationship analysis (See Supplemental Table 7 in Additional file 2). In overall, stable effects were observed in most of the subgroups except for studies conducted in MI (RR: 0.36; 95% CI: 0.10–1.27) and not included diet in LB score (RR: 0.36; 95% CI: 0.07–1.93) which showed that there were no associations in the healthiest vs. least-healthy combination of LBs analysis, and study not included diet in LB score (RR: 0.94; 95% CI: 0.84–1.05) also showed no association in the dose-response relationship analysis (See Supplemental Tables 6–7 in Additional file 2). The pooled estimates remained significant and stable when sensitivity analyses were performed after removing one study at a time (See Supplemental Figs. 2–3 in Additional file 2).

### Association of LBs with CVD mortality in general population

**Fig. 4:**
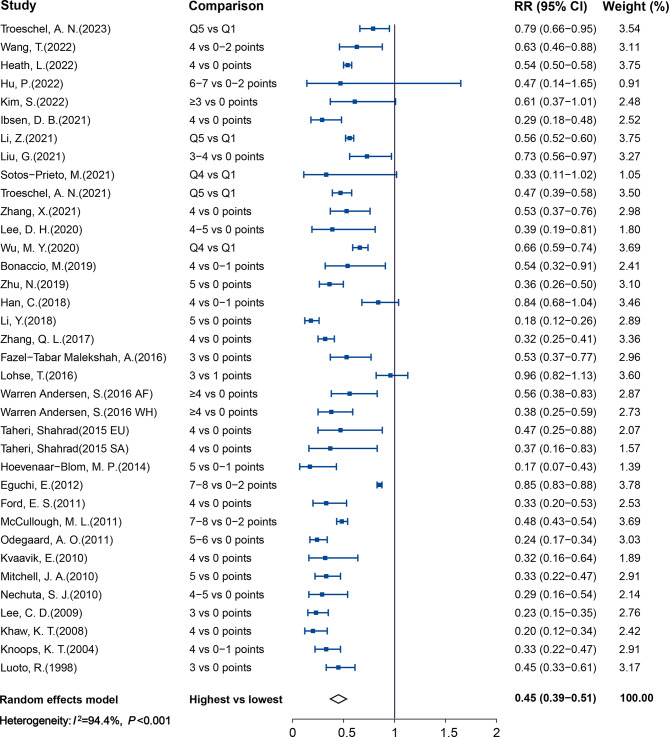
Forest plot of pooled relative risk for CVD mortality with the healthiest versus the least healthy combination of LBs

**Fig. 5:**
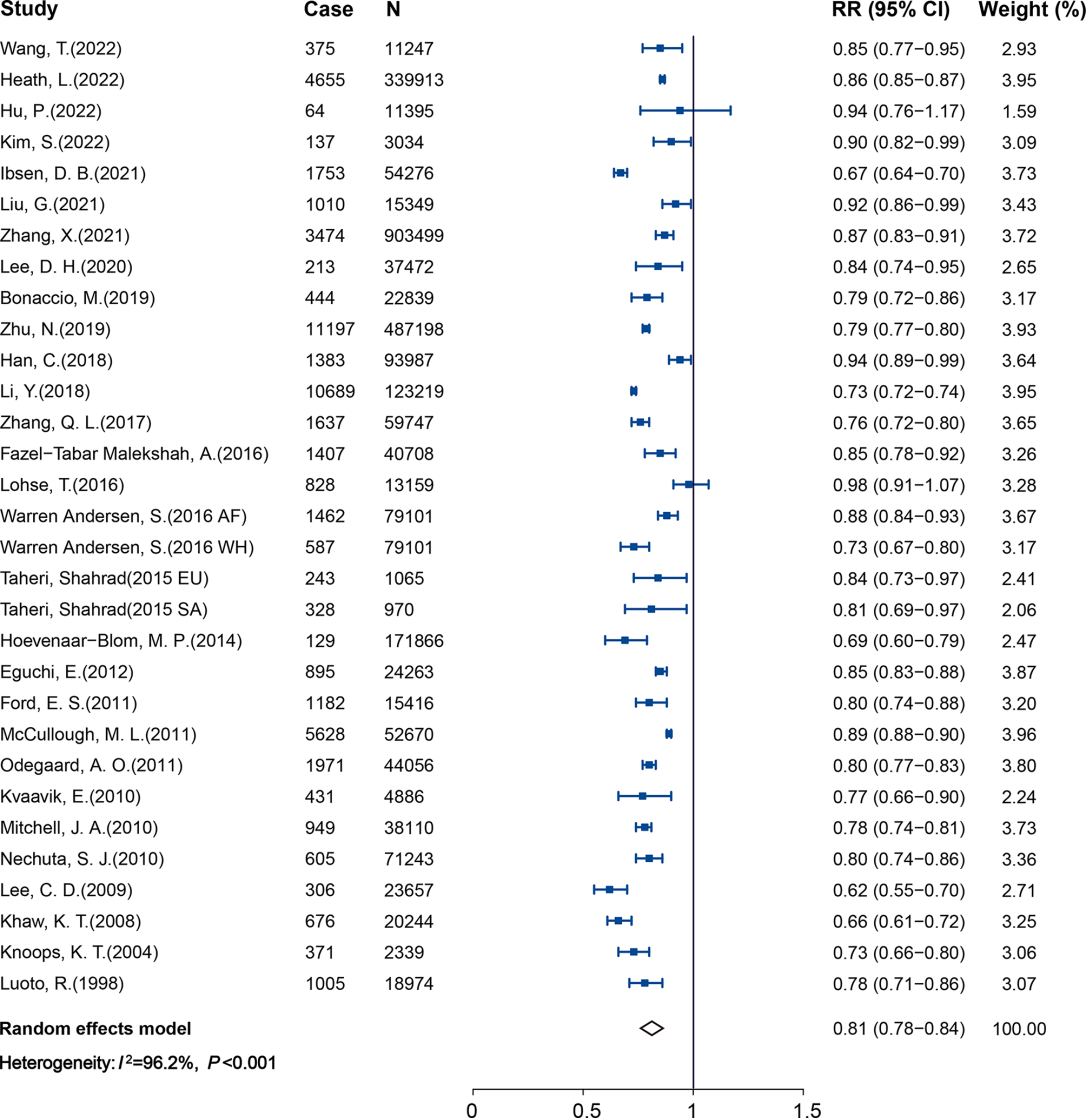
Forest plot for the pooled association between per 1 healthy LB increment and CVD mortality

Figure 4 shows the association between LBs and CVD mortality, with a total of 3,197,553 participants and 68,211 cases. Compared with individuals with the least-healthy combination of LBs, those with the healthiest had a 55% lower risk of CVD mortality (RR 0.45, 95%CI: 0.39–0.51; *I*^2^ = 94.4%, *P*_heterogeneity_ < 0.001). Publication bias was observed by the asymmetrical funnel plot and Egger’s test, but the result was not altered after using the trim- and- fill method to adjust for publication bias (RR: 0.45; 95% CI: 0.39–0.52; See Supplemental Fig. 4A in Additional file 2). 31 studies were included in the dose–response analysis of LBs and CVD mortality with 2,785,902 participants and 56,034 cases (Fig. 5). The pooled RR per 1 healthy LBs increment was 0.81 (95% CI: 0.78–0.84; *I*^2^ = 96.2%, *P*_heterogeneity_ < 0.001). No publication bias was detected by the funnel plot (See Supplemental Fig. 4B in Additional file 2) and Egger’s test (*P* = 0.375).

Meta-regression and subgroup analyses were conducted to explore the potential sources of heterogeneity. Meta-regression indicating that adjustment educational level may explain the high heterogeneity across studies in the healthiest vs. least-healthy combination of LBs, and continent and factors included in combination of LBs with or without smoking may the additional source of heterogeneity in the dose-response relationship analysis (all *P*_regression_<0.05; See Supplemental Tables 8–9 in Additional file 2). The findings from subgroup analyses generally supported the overall findings of the study. However, it is worth noting that the subgroup analysis for studies that did not include smoking in combination with LBs showed no significant association between LBs and CVD mortality in the analysis comparing the healthiest versus least-healthy combination of LBs (RR: 0.63; 95% CI: 0.38–1.05) (See Supplemental Tables 8–9 in Additional file 2). The pooled estimates remained significant and stable when sensitivity analyses were performed after removing one study at a time (See Supplemental Figs. 5–6 in Additional file 2).

### Association between LBs and prognosis among individuals with CVD

Supplemental Fig. 7 in Additional file 2 shows the associations between LBs and CVD recurrence, CVD mortality, and all-cause mortality among individuals with CVD. The pooled RRs comparing participants with the healthiest versus the least-healthy combination of LBs were 0.38 (95%CI: 0.25–0.58; *I*^2^ = 0.0%, *P*_heterogeneity_ =1.000; 4,890 participants and 623 cases) for CVD recurrence and 0.33 (95%CI: 0.15–0.71; *I*^2^ = 86.4%, *P*_heterogeneity_ = 0.001; 465,609 participants and 13,551 cases) for all-cause mortality. In the dose-response analysis of LBs and prognosis among individuals with CVD, the pooled RRs with per 1 healthy LB increase were 0.73 (95%CI: 0.66–0.80; *I*^2^ = 0.0%, *P*_heterogeneity_ =0.608) for CVD recurrence and 0.73 (95%CI: 0.59–0.90; *I*^2^ = 92.1%, *P*_heterogeneity_ <0.001) for all-cause mortality among individuals with CVD (See Supplemental Fig. 8 in Additional file 2). Due to the limited number of studies on CVD recurrence and all-cause mortality, the corresponding meta-regression, subgroup analyses, sensitivity analyses, and publication bias assessments were not performed in the current meta-analysis. In addition, it is noteworthy that only one study reported the association of LBs and CVD mortality among participants diagnosed with CVD and hence the pooled RR could not be performed. In this study [18], individuals exhibiting the healthiest combination of LBs demonstrated a remarkable 92% reduction in the risk of CVD mortality compared to those with the least-healthy combination of LBs. Furthermore, each incremental increase in healthy LBs corresponded to a 47% decrease in the risk of CVD mortality.

## Discussion

The current meta-analysis gathered a total of 29 prospective cohort studies to examine the association between combination of LBs and CVD, 36 studies between LBs and CVD mortality. Meanwhile, 6 prospective cohort studies were gathered to examine the association of LBs and prognosis of individuals with CVD (2 on CVD recurrence, 1 on CVD mortality, and 3 on all-cause mortality). We provided comprehensive and quantitative estimates for the associations between LBs and CVD, CVD mortality, as well as adverse outcomes in CVD individuals after adjustment for confounding factors.

This study indicated that, individuals with the healthiest combination of LBs would have a 58% and 55% lower risk of incident CVD and CVD mortality, respectively. With per 1 healthy LBs increment, the risk of CVD and CVD mortality are decreased by 17% and 19%, respectively. The associations were consistent among populations with most diverse socioeconomic backgrounds and baseline characteristics. Moreover, adopting a healthy LB was associated with a 62% and 67% lower risk of CVD recurrence and all-cause mortality among individuals diagnosed with CVD. Additionally, for each incremental increase in healthy LBs, the risk of CVD recurrence and all-cause mortality decreased by 27% and 27%, respectively.

There has not been a meta-analytical synthesis of LBs with the risk of CVD, CVD mortality, and the prognosis of individuals with CVD to date, though several meta-analyses addressed the associations between lifestyle indices and the risk of CVD and mortality. One meta-analysis comprising five LBs (physical activity, smoking, diet, alcohol consumption, and body weight) [90] reported consistent results with our study. It was found that a healthy LB was associated with a reduced risk of 66% for CVD, 60% for stroke, and 69% for HF. Another meta-analysis concluded that adopting the healthy lifestyle was associated with a 62% and 58% reduced risk for CVD and CVD mortality, and a 55–71% lower risk of multiple subtypes of CVDs [91]. Unlike to previous articles included metabolic factors such as blood lipids and blood glucose, our meta-analysis included prospective cohort studies purely on LBs. Additionally, our meta-analysis confirmed the benefits of each additional healthy LB increament in lowering the risk of CVD, CVD mortaliy, and adverse outcomes among individuals with CVD. Obviously, the current meta-analysis for the first time represented the comprehensively quantitative correlations between LBs and CVD and the prognosis of the clinical treatment of CVD.

In order to identify potential sources of heterogeneity, we conducted meta regression and various subgroup analyses on the relationships between LBs and CVD and CVD mortality. The results were consistent with the overall findings across different age groups, genders, geographic regions, and adjustment for age, ecomomic level, and educational level, which may have important public health implications, suggesting that people with different demographic characteristics can obtain health benefits by adopting LBs to achieve the purpose of preventing CVD. Evidence found here implied that primary health care service providers should prioritize the assessment of LBs in lowering the risk of CVD [92].

Educational level was found to be an important confounder in the relationship between LB and CVD morbidity and mortality. Slightly different risk relative reductions were found in studies between adjusted and unadjusted educational levels of the current meta-analysis. In general, the population with lower level of education showed worse adherence to healthy LBs. While the causes remain controversial, a significant proportion of previous studies have suggested that poor health awareness and a lack of awareness among individuals may be related to the observed correlations [51]. The inclusion of alcohol consumption as a LB in the LB score could potentially contribute to the high heterogeneity observed in the analysis. It is worth noting that the heterogeneity in subgroups that did not include alcohol consumption as a LB was lower than the overall heterogeneity in the context of CVD.

Correlations were not found between LBs and MI based on the included three prospective cohort studies, including Ford, E. S. et al. (2009) [69], Akesson, A. et al. (2014) [64], and Zuo, Y. et al. (2022) [15]. According to the included study by Cardi, M. et al. (2009) [81], a meaningful finding was concluded that LBs showed no association with CVD when diet was not included into the combinations. The possible reason is that a small number of studies are included, which may have been underpowered to detect associations with adverse outcomes [16, 93].

Our study also added important evidence to a clinical issue that patients with CVD can also benefit from LBs. The findings showed that LBs contribute more protection to individuals with CVD than general population. Participants with the healthiest combination of LBs were related to a reduced of 62% for CVD recurrence and 67% for all-cause mortality among individuals with CVD. Meanwhile, the risk of CVD and CVD mortality in the general population decreased by 58% and 55%, respectively. The risk reductions indicated that LBs modifications are still meaningful and should be recommended for individuals with CVD. As shown in the study by Jeong et al. (2019) [94], individuals with CVD may benefit more from physical activities than the group without CVD. Re-understanding and evaluation of the potential value of LBs in the clinical treatment of CVD, basing on more clinical randomized controlled trials or large-scale prospective cohort studies, has become an urgent task for the global response to the increasing incidence and disease burden of CVD.

Our findings suggest that each additional healthy LB is associated with reductions ranging from 17 to 27% in risk of CVD, CVD mortality, and prognosis. Several other studies have reported the dose-response relationship between LBs and CVD and its prognosis [16, 17, 37, 42, 95]. In a study based on two large prospective Study (Nurses’ Health Study and Health Professionals Follow-up Study; *n* = 121,700), there was a 20% stepwise risk reduction of CVD mortality for each additional healthy LB over 27 follow-up years [96]. Besides, our results also show that the protective effect of the healthiest combination of LBs on CVD and its prognosis is greater compared to individuals with least-healthy combination of LBs. For the individuals with the best quantified combination of LBs, CVD risk was reduced by 58%, CVD mortality was reduced by 55%, and the risk of poor prognosis was reduced by 62–67%, compared with the rest of the population. At the same time, for each additional LB, the risk of CVD decreased by 17%, CVD mortality decreased by 19%, and the risk of CVD poor prognosis decreased by around 27%, respectively. This indicates that the healthiest combination of multiple LBs has a more significant protective effect than simply strengthening a LB.

How LBs affect CVD and its poor prognosis has been partially revealed by some previously published studies. According Warburton, D. E. R. et al. (2017) [97], Lloyd-Price, J. et al. (2016) [98], and Jha, P. et al. (2014) [99], physical activity reduce the risk of chronic diseases through lowering blood pressure, blood sugar and cholesterol. Diet matters with the immune system and metabolic function by affecting the intestinal flora. While smoking increases the risk of adverse outcomes through genetic mutations has been confirmed.

### Strengths and limitations

This is the first systematic review and meta-analysis to summarise the existing dose-response relationships between LBs and CVD in general population, as well as the risk of recurrence, mortality and all-cause mortality among individuals with CVD. Compared to the previous meta-analysis, this study confirmed for the first time that the combination of multiple healthy LBs has a more significant protective effect than simply strengthening a LB. Meanwhile, the constructions of lifestyle scores varied across studies, but this study only included articles containing LBs and did not involve any biochemical attributes such as blood lipids, blood glucose and so on. These LBs are closely related to the primary prevention of CVD and the management of its prognosis and are more conducive to basic public health service providers to assess the level of healthy lifestyles in the population and clinicians to develop comprehensive healthy lifestyle intervention strategies for patients.

There are several limitations in the current meta-analysis that have to be acknowledged. First, the study obtained a limited number of prospective cohort studies available specifically focusing on the prognosis of individuals with CVD. This limitation hindered our ability to perform further stratified analyses and examine the potential effects of LBs on improving the poor prognosis of CVD. Second, the LBs in all included studies were self-reported, meaning the validity of the study may be limited as it can be argued that the data represent only a collection of memories or subjective perceptions of lifestyle behaviors. Third, it should be noted that there is always the variability in the selection of confounding variables for adjustment across different studies. This variations in the choice and inclusion of confounders may have introduced residual confounding, which could not be completely ruled out. However, we conducted subgroup analysis based on whether common confounding factors were adjusted, and found that the most results were consistent with the main findings. Fourth, it is essential to acknowledge that the distinguishment of the priority of different LBs and the establishment of specific thresholds or ranges for each LB were not feasible due to the unavailability of individual-level original data. Instead, all LBs are treated to have equally significant contributions and the division of thresholds or ranges relies exclusively on the definitions provided by the respective authors of the included studies. It is noteworthy that these methodologies align with established practices observed in prior meta-analyses in the same research domain^100–102^. Lastly, it is important to acknowledge that there was moderate to high statistical heterogeneity observed in most of the analyses. Previous evidence has shown that there is substantial heterogeneity in the estimation of correlation in most analyses. We performed meta regression and subgroup analysis to explore the sources of heterogeneity, the findings suggest that the composition of LBs, confounding factors of adjustment and sub-types of outcomes may be the potential sources of heterogeneity.

## Conclusions

LBs are associated with substantial risk reduction in CVD, CVD mortality, and adverse outcomes among individuals with CVD. Meanwhile, the combination of multiple healthy LBs has a more significant protective effect on CVD compared to merely focusing on strengthening a single LB. Multiple LBs, instead of tackling one certain LB, should be recommended for the prevention and management of CVD. With the growing incidence and burden of CVD globally, there is an urgent need to pay more attention to the role of LBs in individuals with CVD in the future, providing evidence for the prevention and clinical treatment of adverse outcomes in patients with CVD.

### Electronic supplementary material

Below is the link to the electronic supplementary material.

Supplementary Material 1

Supplementary Material 2

Supplementary Material 3

## Data Availability

Data (including the extracted contents from the searched articles) are available upon reasonable request from Dr. Yifei Feng; mail: fengyifei2019@163.com.

## Abbreviations

CVD: Cardiovascular disease
CHD: Coronary heart disease
CI: Confidence interval
HF: Heart failure
HR: Hazard ratio
IHD: Ischemic heart disease
LB: Lifestyle behavior
MI: Myocardial infarction
OR: Odds ratio
PRISMA: Preferred Reporting Items for Systematic Reviews and Meta-Analyses
PROSPERO: Prospective Register of Systematic Reviews
RR: Relative risk
