# Supplementary material for: Lifestyle behaviors and risk of cardiovascular disease and prognosis among individuals with cardiovascular disease: a systematic review and meta-analysis of 71 prospective cohort studies
Source: Int J Behav Nutr Phys Act. 2024 Apr 22;21:42. doi: 10.1186/s12966-024-01586-7 (PMC11036700; doi:10.1186/s12966-024-01586-7)
Supplement: Supplementary file 3 — Supplementary Material 3 [file 12966_2024_1586_MOESM3_ESM.doc]

**Supplemental Materials**

**Supplemental Table 1.** Systematic literature review search terms and strategy.

**Supplemental Table 2.** Characteristics of studies related to the incidence of CVD.

**Supplemental Table 3.** Characteristics of studies related to CVD mortality.

**Supplemental Table 4.** Characteristics of studies related to CVD recurrence, mortality and all-cause mortality among individuals with CVD.

**Supplemental Table 5.** Assessment of quality of included studies (Newcastle-Ottawa Quality Assessment Scale).

**Supplemental Table 6.** Subgroup analyses of LBs and risk of CVD.

**Supplemental Table 7.** Subgroup analyses of per 1 healthy LB increment and risk of CVD.

**Supplemental Table 8.** Subgroup analyses of LBs and risk of CVD mortality.

**Supplemental Table 9.** Subgroup analyses of per 1 healthy LB increment and risk of CVD mortality.

**Supplemental Figure 1.** Funnel plots for assessing the association between LBs and CVD (after the trim and fill analysis; A, the healthiest versus the least-healthy combination of LBs; B, per 1 healthy LB increment).

**Supplemental Figure 2.** Sensitivity analyses estimates (RR with corresponding 95% CI) for the associations between LBs and CVD.

**Supplemental Figure 3.** Sensitivity analyses estimates (RR with corresponding 95% CI) for the associations between per 1 healthy LB increment and CVD.

**Supplemental Figure 4.** Funnel plots for assessing the association between LBs and CVD mortality (A, the healthiest versus the least-healthy combination of LBs, after the trim and fill analysis; B, per 1 healthy LB increment).

**Supplemental Figure 5.** Sensitivity analyses estimates (RR with corresponding 95% CI) for the associations between LBs and CVD mortality.

**Supplemental Figure 6.** Sensitivity analyses estimates (RR with corresponding 95% CI) for the associations between per 1 healthy LB increment and CVD mortality.

**Supplemental Figure 7.** Forest plot of pooled relative risk for CVD recurrence, mortality and all-cause mortality among individuals with CVD with the healthiest versus the least-healthy combination of LBs.

**Supplemental Figure 8.** Forest plot for the pooled associations between per 1 LB increment and CVD recurrence, mortality and all-cause mortality among individuals with CVD.

**Supplemental Table 1.** Systematic literature review search terms and strategy.

| **Search terms for PubMed** |
| --- |
| #1 “life style”[Mesh] OR “risk reduction behavior”[Mesh] OR “health behavior”[Mesh] OR “life style”[Title/Abstract] OR “life styles”[Title/Abstract] OR “health factor”[Title/Abstract] OR “health factors”[Title/Abstract] OR “lifestyle”[Title/Abstract] OR “lifestyles”[Title/Abstract] OR “protective factor”[Title/Abstract] OR “protective factors”[Title/Abstract] OR “risk reduction behavior”[Title/Abstract] OR “risk reduction behaviour”[Title/Abstract] OR “risk reduction behaviors”[Title/Abstract] OR “risk reduction behaviours”[Title/Abstract] OR “health behavior”[Title/Abstract] OR “health behaviour”[Title/Abstract] OR “health behaviors”[Title/Abstract] OR “health behaviours”[Title/Abstract] OR “healthy behavior”[Title/Abstract] OR “healthy behaviour”[Title/Abstract] OR “healthy behaviors”[Title/Abstract] OR “healthy behaviours”[Title/Abstract] OR “risk behavior”[Title/Abstract] OR “risk behaviour”[Title/Abstract] OR “risk behaviors”[Title/Abstract] OR “risk behaviours”[Title/Abstract] OR “modifiable factors”[Title/Abstract] |
| #2 “combination”[Title/Abstract] OR “combinations”[Title/Abstract] OR “combined”[Title/Abstract] OR “composite”[Title/Abstract] OR “integrated”[Title/Abstract] OR “interaction”[Title/Abstract] OR “interactions”[Title/Abstract] OR “joint effect”[Title/Abstract] OR “joint effects”[Title/Abstract] OR “merged effect”[Title/Abstract] OR “merged effects”[Title/Abstract] OR “score”[Title/Abstract] OR “scores”[Title/Abstract] OR “adherence to”[Title/Abstract] OR “adhere to”[Title/Abstract] OR “adhered to”[Title/Abstract] OR “collective”[Title/Abstract] OR “cumulative”[Title/Abstract] OR “multiple”[Title/Abstract] |
| #3 “cerebrovascular disorders”[MeSH] “cardiovascular Diseases”[MeSH] OR “cardiovascular disease”[Title/Abstract] OR “cerebrovascular disorders”[Title/Abstract] OR “cardiovascular diseases”[Title/Abstract] OR “CVD”[Title/Abstract] OR “coronary disease”[Title/Abstract] OR “coronary artery disease”[Title/Abstract] OR “coronary heart disease”[Title/Abstract] OR “CHD”[Title/Abstract] OR “ischemic heart disease”[Title/Abstract] OR “ischemic heart disease”[Title/Abstract] OR “stroke”[Title/Abstract] OR “cerebrovascular disease”[Title/Abstract] OR “cerebrovascular disorders”[Title/Abstract] OR “heart disease”[Title/Abstract] OR “myocardial infarction”[Title/Abstract] OR “MI”[Title/Abstract] OR “heart failure”[Title/Abstract] OR “cerebral vascular accident”[Title/Abstract] OR “CVA”[Title/Abstract] OR “cardiovascular”[Title/Abstract] OR “coronary”[Title/Abstract] OR “myocardial”[Title/Abstract] |
| #4 “prospective studies”[Mesh] OR “cohort studies”[Mesh] OR “longitudinal studies”[Mesh] OR “prospective”[Title/Abstract] OR “longitudinal”[Title/Abstract] OR “cohort”[Title/Abstract] OR “cohorts”[Title/Abstract] OR “follow-up”[Title/Abstract] OR “followed up”[Title/Abstract] OR “observational Study”[Mesh] OR “Observational Study”[Title/Abstract] |
| #5 (“rats”[Mesh] OR “mice”[Mesh] OR “rat”[Title/Abstract] OR “rats”[Title/Abstract] OR “mouse”[Title/Abstract] OR “mice”[Title/Abstract] OR “vivo”[Title/Abstract] OR “vitro”[Title/Abstract]) |
| (#1 AND #2 AND #3 AND #4) NOT #5 |
| **Search terms for Embase 13065 2/10** |
| #1 life style.mp. or lifestyle/ or risk reduction behavior.mp. or risk reduction/ or health behavior.mp. or health behavior/ or life styles.mp. or health factor.mp. or health factors.mp. or healthy lifestyle/ or lifestyle modification/ or lifestyles.mp. or protective factor.mp. or protective factors.mp. or risk reduction behaviour.mp. or risk reduction behaviors.mp. or health behaviour.mp or health behaviors.mp. or healthy behavior.mp. or risk behavior.mp. or risk behavior/ or modifiable factors.mp. |
| #2 combination.mp. or combinations.mp. or combined.mp. or composite.mp. or integrated.mp. or interaction.mp. or interactions.mp. or joint effect.mp. or joint effects.mp. or merged effect.mp. or merged effects.mp. or score.mp. or scores.mp. or adherence to.mp. or adhere to.mp. or adhered to.mp. or collective.mp. or cumulative.mp. or multiple.mp. |
| #3 cerebrovascular disorders.mp. or cerebrovascular disease/ or cardiovascular diseases.mp. or cardiovascular disease/ or coronary heart disease.mp. or heart disease.mp. or heart disease/ or ischemic heart disease.mp. or ischaemic heart disease.mp. or ischemic heart disease/ or coronary artery disease/ or CHD.mp. or myocardial infarction.mp. or heart infarction/ or stroke.mp. or cerebrovascular accident/ or ischemic stroke.mp. or brain ischemia/ or haemorrhagic stroke.mp. or brain hemorrhage/ or hemorrhagic stroke.mp. or brain hemorrhage/ or CVD.mp. or coronary disease.mp. or heart failure.mp. or heart failure/ or cerebral vascular accident.mp. or cerebrovascular accident/ or cardiovascular.mp. or coronary.mp. or myocardial.mp. |
| #4 cohort studies.mp. or cohort analysis/ or prospective studies.mp. or prospective study/ or longitudinal studies.mp. or longitudinal study/ or follow-up studies.mp. or follow up.mp. or follow up/ or cohort.mp. or prospective.mp. or longitudinal.mp. |
| (#1 AND #2 AND #3 AND #4) limits to human |
| **Search terms for Web of Science** |
| #1 TS= (“life style” OR “risk reduction behavior” OR “health behavior”OR “life styles”OR “health factor” OR “health factors” OR “lifestyle” OR “lifestyles” OR “protective factor” OR “protective factors” OR “risk reduction behaviour” OR “risk reduction behaviors”OR “risk reduction behaviours” OR “health behaviour” OR “health behaviors” OR “health behaviours” OR “healthy behavior” OR “healthy behaviour” OR “healthy behaviors” OR “healthy behaviours” OR “risk behavior” OR “risk behaviour”OR “risk behaviors”OR “risk behaviours” OR “modifiable factors”) |
| #2 TS= (“combination” OR “combinations” OR “combined” OR “composite” OR “integrated” OR “interaction” OR “interactions” OR “joint effect” OR “joint effects” OR “merged effect” OR “merged effects” OR “score” OR “scores” OR “adherence to” OR “adhere to” OR “adhered to” OR “collective” OR “cumulative” OR “multiple”) |
| #3 TS= (“cerebrovascular disorders” OR “cardiovascular diseases” OR “cardiovascular disease” OR “CVD” OR “coronary disease” OR “coronary artery disease” OR “coronary heart disease” OR “CHD” OR “ischemic heart disease” OR “stroke” OR “cerebrovascular disease” OR “heart disease” OR “myocardial infarction” OR “MI” OR “heart failure” OR “cerebral vascular accident” OR “CVA” OR “cardiovascular” OR “coronary” OR “myocardial”) |
| #4 TS= (“prospective” OR “longitudinal” OR “cohort” OR “cohorts” OR “follow-up” OR “followed up” OR “observational study”) |
| #5 TS= (“rats” OR “mice” OR “rat” OR “mouse” OR “vivo” OR “vitro”) |
| (#1 **AND** #2 AND #3 AND #4) NOT #5 |

**Supplemental Table 2.** Characteristics of studies related to incident CVD.

| **Author (year)** | **Cohort** | **Definition of LBs** | **Score category** | **Outcome attainment** | **Adjustments** |
| --- | --- | --- | --- | --- | --- |
| Mo, M. (2023) | The Stockholm Public Health Cohort | Alcohol drinking: 0. At least 1 time/m; 1. 1–6 times/y; 2. Never. BMI: 0.>=30 kg/m^2^; 1. 25-29.9 kg/m2; 2.<25 kg/m2. Smoking: 0. Current smoking; 1.Former smoking, quit smoking<=1 year; 2.Never smoking or quit >1 year. PA: 0. No regular physical activity; 1. Walking/cycling less than 20 min/day or exercise <=2 h/week; 2.Walking/cycling >20 min/day or exercise >2 h/week. Diet: 0. Fruits consumption less than once a week; 1. Fruits consumption a few times per week; 2. Fruits consumption every day. | <=4 scores 5-6 scores >=7 scores | Extracted from the Swedish National Patient Register and from the Cause of Death Register. | Age, sex, education, drinking and maternal CVD |
| Mao, Ziling (2023) | The REasons for Geographic and Racial Differences in Stroke | Alcohol drinking: 1.>=7.5 drinks/wk (man) or >=6.0 drinks/wk (woman); 2. 3.0–7.0 drinks/wk (man) or 2.0–5.0 drinks/wk(woman); 3. 1.0–3.0 drinks/wk (man) or 0.5–1.75 drinks/wk (woman); 4. > 0.0–< 1.0 drinks/wk (man) or > 0.0–< 0.5 drinks/wk (woman); 5. 0 drink/wk. Sedentary behaviour: 1. >=4hrs./d; 2. 3hrs./d; 3. 2hrs./d; 4. 1 hrs./d; 5. < hrs./d. Smoking: 1. >=35.0 pack-yrs; 2. 16.0–34.9 pack-yrs; 3. 4.5–15.pack-yrs; 4. < 4.5 pack-yrs; 5. 0pack-yrs. WC:>=109.0cm(man) or>=104.2cm(woman); 101.6–108.9 cm(man) or 94.1–104.1 cm(woman); 95.9–101.5 cm(man) or 86.1–94.0 cm(woman); 89.0–95.8 cm(man) or 78.6–86.0cm(woman); 60.9–88.9 cm(man) or 55.9–78.5cm(woman). Diet (evolutionary-concordance C score): 1. 17–33 scores; 2. 34–37 scores; 3. 38–40 scores; 4. 41–44 scores; 5. 45–60 scores. PA: 1. 0 times/wk; 2. 1–2 times/wk; 3. 3–4 times/wk; 4. 5–6 times/wk; 5. >=7 times/wk. Social participation: 1. 0–5people; 2. 6–7 people; 3. 8–11 people; 4. 12–17 people; 5. >=18 people. | 6–15 scores 16–17 scores 18–19 scores 20–21 scores 22–30 scores | Ascertained through telephone with participants or their designated proxies and adjudicated by medical records, death certificates, autopsy reports and a committee of trained adjudicators. | Age, race, income, education status, health insurance, sex/postmenopausal hormone use, statin use, baseline SBP and DBP, region, history of diabetes mellitus, history of hypertension, history of cancer, history of kidney failure, regular aspirinuse, regular non-aspir in NSAID use, total energy intake, family history of CVD in a first-degree relative. |
| Zuo, Y. (2022) | The Kailuan study | Smoking: 0. not current smokers; 1. current smokers. Alcohol drinking: 0. not current drinking; 1. current drinking. PA: 0. >=80 min/wk; 1. < 80 min/wk; 2. None. Sedentary behavior: 0. < 4h/d; 1. 4–8 h/d; 2. >=8 h/d. Diet: 0. salt intake<6g/day; 1. salt intake 6–10g/day; 2. salt intake>=10g/day. | 0-1 scores 2 scores 3-5 scores | Ascertained through Municipal Social Insurance, Hospital Discharge Register, medical records and vital statistical offices. | Age, sex, BMI, education and family income at baseline. |
| Heath, L. (2022) | The UK Biobank | Smoking: 0. current or former smoking; 1. never smoker with 0 cigarettes/ day throughout life. Alcohol drinking: 0. >=112 g/week; 1. <112 g/week. Diet: 0. <5 servings of fruit and vegetable/day; 1. 5 or more servings of fruit and vegetable/day. PA: 0. <150 min/week of moderate-vigorous activity; 1. 150 min/week or more of moderate-vigorous activity. | 0 score 1 score 2 scores 3 scores 4 scores | Identified by hospital admission data in England and death registries. | Age, sex, ethnicity, Townsend Deprivation Score, education, region, family history of CVD, family history of diabetes, menopausal status, and BMI group. |
| Guasch-Ferré, Marta (2022 NHS) | The Nurses’ Health Study | Smoking: 1. not currently smoking; 0. currently smoking. PA: 1. moderate to vigorous intensity exercise >=3.5 h/wk; 0. moderate to vigorous intensity exercise <3.5 h/wk. Diet (AHEI): 1. diet score in the top 40%; 0. diet score in the surplus part. BMI: 1. 18.5-24.9 kg/m^2^; 0. <18.5 or>24.9 kg/m^2^. Alcohol drinking: 1. 5-14.9 grams/d for women and 5-30 grams/d for men; 0. <5 or >14.9 grams/d for women and <5 or >30 grams/d for men. Sleep duration: 1. 6-8 hours/day as per the distribution;0. <6 or >8 hours/day as per the distribution. | 0 score 1 score 2 scores 3 scores 4 scores 5 scores 6 scores | Identified by reports of families, the US postal system, or death certificates from state vital-statistics departments and the National Death Index and confirmed through a review of medical records or autopsy reports. | Age; ethnicity; current multivitamin and aspirin use; family history of diabetes mellitus, MI, or cancer; and menopausal status and hormone use. |
| Guasch-Ferré, Marta (2022 HPFS) | The Health Professionals Follow-up Study | Smoking: 1. not currently smoking; 0. currently smoking. PA: 1. moderate to vigorous intensity exercise >=3.5 h/wk; 0. moderate to vigorous intensity exercise <3.5 h/wk. Diet (AHEI): 1. diet score in the top 40%; 0. diet score in the surplus part. BMI: 1. 18.5-24.9 kg/m^2^; 0. <18.5 or>24.9 kg/m^2^. Alcohol drinking: 1. 5-14.9 grams/d for women and 5-30 grams/d for men; 0. <5 or >14.9rams/d for women and <5 or >30 grams/d for men. Sleep duration:1. 6-8 hours/day as per the distribution;0. <6 or >8 hours/day as per the distribution. | 0 score 1 score 2 scores 3 scores 4 scores 5 scores 6 scores | Identified by reports of families, the U.S. postal system, or death certificates from state vital-statistics departments and the National Death Index and confirmed through a review of medical records or autopsy reports. | Age; ethnicity; current multivitamin and aspirin use; family history of diabetes mellitus, MI, or cancer. |
| Yang, R. (2021) | The China Kadoorie  Biobank | Smoking: 1. non-current smokers and occasional smokers; 0. >=1cig/d. Alcohol drinking: 1. <=25 g/d in men and <=15 g/d in women; 0. >25 g/d in men and >15g/d in women. PA: 1. above median; 0. below median. Diet: 1. vegetables and fruit daily and red meat 1–6 d/wk; 0. vegetables and fruit not daily and red meat < 1–6 d/wk. Obesity: 1. 18.5–23.9 kg/m^2^ and WC <90 cm in men and <85 cm in women; 0. <18.5 or >23.9 kg/m^2^ and WC >=90 cm in men and >=85 cm in women. | <=1 score 2 scores 3 scores >=4 scores | Identified through electronic linkage to disease and death registries and national health-insurance databases, with coded records of all hospitalization events and procedures. | Sex, education, marital status, menopausal status (only in women) and family history of heart attack or stroke at baseline, and stratified jointly by study area and age at baseline in 5-year intervals |
| Han, Y. (2021) | The China Kadoorie  Biobank | Smoking: 0. current smokers and former smokers who quit because of illness to the high-risk group; 1. not current smokers. Alcohol drinking: 0. >=30 g/d or having stopped drinking; 1. 0-30 g/d. Diet: 0. non-daily eating of vegetables, fruits, and eggs, and eating red meat daily or less than weekly. 1. daily eating of vegetables, fruits, and eggs, and eating red meat daily or more than weekly.  PA: 0. below the mediate; 1. above the mediate. Body shape: 0. BMI<18.5 or >=28.0 kg/m^2^ or having WC >=90 cm (men)/85 cm (women); 1. BMI 18.5-28.0kg/m^2^ or having WC<90 cm (men)/85 cm (women) | 0 score 1 score 2 scores 3 scores 4 scores 5 scores | Identified through  linkages to disease and mortality registries and national health insurance claim database, supplemented with local residential records and annual active confirmation. | Age, study area, sex, education, marital status, parental family history of cardiometabolic multimorbidity. |
| Tsai, Ming-Chieh (2021) | The 2002 Taiwan Survey of Hypertensive, Hyperglycemia, Hyperlipidemia Survey | BMI: 1. <25 kg/m^2^; 0. >=25 kg/m^2^. Diet: 1. Mediterranean diet>=6 points; 0. Mediterranean diet<6 points. PA: 1. 1–150 min/k; 0. 0 or>150 min/week. Smoking: 1. never smoking; 0. current or quit smoking. Alcohol drinking: 1. frequent drinking; 0. less or no drinking. | 0-1 score 2 scores 3 scores 4-5 scores | Obtained from the National Health Insurance Research Database and Taiwan Cause of Death Register. | Age, sex, education, average month income, marital status, parental history of CVD, menopause status and estrogen exposure, baseline hypertension, baseline diabetes mellitus, history of hyperlipidemia, SBP, DBP, triglyceride, non-HDL, fasting glucose, HbA1c. |
| Dimovski, K. (2019) | The Malmö Diet  and Cancer study | Smoking:1. no current smoking; 0. current smoking. Obesity: 1. BMI <30kg/m^2^; 0. BMI >=30 kg/m^2^. PA: 1. >=1times/wk; 0. <1times/wk. Diet: 1. Healthy diet; 0. unhealthy diet. | 0-1 score 2 scores 3-4 scores | Identifed through linkage of the personal identification number with three registers: the Swedish Hospital Discharge register, the Swedish Cause of Death Register, and the Swedish Coronary Angiography and Angioplasty Registry. | Age, sex, educational level, and parental history of MI. |
| Diaz-Gutierrez, J. (2018) | The SUN project | Smoking: 1. exsmoker; 0. active smoker. PA: 1. > 20MET-h/wk; 0. <=20MET-h/wk. Mediterranean diet: 1. 4 adherence points; 0. <4 adherence points. BMI: 1. <=22 kg/m^2^; 0. >22 kg/m^2^. Alcohol drinking: 1. 0.1-5.0 g/d for women and 0.1-10.0g/d for men; 0. <0.1or>5.0 g/d for women and <0.1or>10.0 g/d for men. TV: 1. < 2 h/d; 0. >=2 h/d. Alcohol: 1. <=5 alcoholic drinks at anytime; 0. >5 alcoholic drinks  Afternoon nap: 1. <30 min/d; 0. >=30 min/d. Meet friends: 1. > 1 h/d; 0. <1 h/d. Work time: 1. >=40 h/wk; 0. < 40 h/wk. | 0-3 scores 4 scores 5 scores 6 scores 7-10 scores | Identified through medical records and reports with the permission of participants’ next-of-kin. | Sex, age, year of questionnaire completion, diabetes, CVD, hypertension, hypercholesterolemia, and hypertriglyceridemia. |
| Lv, J. (2017) | The China Kadoorie  Biobank | Smoking: 1. nonsmokers or those who had stopped smoking for reasons other than illness for >=6months; 0. current smokers. Alcohol drinking: 1. 0-30g/d; 0. None or >30g/d. PA: 1. >=20MET-h/d for men and >=18 MET-h/d for women; 0. <20MET-h/d for men and <18 MET-h/d for women. Diet: 1. vegetables and fruits daily and red meat 1 to 6d/wk; 0. vegetables and fruits not daily and red meat <1 or >6d/wk. BMI: 1. 18.5- 23.9 kg/m^2^; 0. <18.5 or >23.9 kg/m^2^. WHR: 1. <0.90 in men and <0.85 in women; 0.>= 0.90 in men and >=0.85 in women. | 0 score 1 score 2 scores 3 scores 4 scores >=5 scores | Identified by using linkage with local disease and death registries, with the recently established national health insurance system, and by active follow-up. | Age, sex, education, marital status, family histories of heart attack or stroke, and prevalent hypertension at baseline. |
| Larsson, S. C. (2016) | The Cohort of Swedish Men and Swedish Mammography  Cohort | Smoking: 1. nonsmoker; 0. current smoker. PA: 1. >=150 min/wk; 0. <150 min/wk. Diet: 1. vegetables and fruits daily and red meat 1 -6d/wk; 0. vegetables and fruits not daily and red meat <1 or >6d/wk. BMI: 1. 18.5- 25 kg/m^2^; 0. <18.5 or >25 kg/m^2^. | 0 score 1 score 2 scores 3 scores 4 scores | Ascertained by linkage with the  Swedish National Patient Register and the Swedish Cause of Death Register. | Age, education, family history of MI before age 60 years, aspirin use, and history of hypertension, hypercholesterolemia, diabetes mellitus, and atrial fibrillation. |
| Chomistek, A. K. (2015) | The Nurses’ Health Study II | Smoking: 1. not currently smoking; 0. currently smoking. PA: 1.>=2.5 h/wk; 0.<2 .5 h/wk. TV: 1. <=7 h/k; 0.>7 h/k. BMI: 1. 18.5 -24.9 kg/m^2^; 0. <18.5 or >24.9 kg/m^2^. Alcohol drinking: 1. 0.1 to 14.9 g/d; 0. <0.1 or >14.9g/d. Diet: 1. Index–2010 (AHEI-2010) score in the top 40%; 0. Index–2010 (AHEI-2010) score in the last 60%. | 0 score 1 score 2 scores 3 scores >=4 scores | Confirmed by hospital or autopsy records or the death certificate and evidence of previous CHD. | Age, time period, parental history of MI before 60 years of age, aspirin use, menopausal status, postmenopausal hormone use, parity, and oral contraceptive use, history of hypertension or hypercholesterolemia at baseline. |
| Del Gobbo, L. C. (2015) | The Cardiovascular  Health Study | Diet: 1. lower 2 quintiles; 0. upper 3 quintiles. PA: 1. walking pace >=2 mph and leisure activity >=850 kcal/wk; 0. walking pace <2 mph and leisure activity <850 kcal/wk. Alcohol drinking: 1. >=1 drink/wk; 0. < 1 drink/wk. Smoking: 1. no current smoking; 0. current smoking. Obesity: 1. BMI<30 kg/m2; 0. BMI>=30 kg/m^2^. | 0-1 score 2 scores 3 scores 4 scores 5 scores | Adjudicated by a centralized events committee using outpatient and inpatient medical records, diagnostic tests, clinical consultations, and interviews. | Age, sex, race, enrollment site, education, and income. |
| Akesson, A. (2014) | The population-based, prospective cohort of Swedish men | Smoking: 1. no smoking; 0. the rest. PA: 1. >=40 min/d walking/bicycling and >= 1 h/wk exercise; 0. < 40 min/d walking/bicycling and <1 h/wk exercise. Abdominal adiposity: 1. <95 cm WC; 0.>=95cm WC. Alcohol consumption: 1.10–30 g/day; 0.the rest. Diet:1. Recommended Food Score top quintile; 0. the rest. | 0 score 5 scores | Ascertained from Swedish National Inpatient and Cause of Death Registers and baseline. | Age, educational achievement, family history of MI, use of aspirin, marital status, non-Recommended Food Score, and total energy intake. |
| Agha, G. (2014) | The Women’s Health Initiative observational study | Diet: 1. high-scoring AHEI (quintiles 4 and 5); 0. low-scoring AHEI (quintiles 1, 2, 3). PA: 1. physically active; 0. somewhat active or inactive. BMI: 1. >=18.5 and <25 kg/m^2^; 0. >=25 kg/m^2^. Smoking: 1. past or never smoker; 0. current smoker. | 0 score 1 score 2 scores 3 scores 4 scores | Ascertained yearly in Women’s Health Initiative by medical record abstraction of self-reported hospitalizations and classified by trained adjudicators using standardized methodology. | Age, race/ethnicity, marital status, education, U.S. region, and antecedent coronary heart disease, treated diabetes, and hypertension. |
| Larsson, S. C. (2014) | The Swedish Mammography Cohort | Diet: 1. score >=21; 0. score <21. Alcohol drinking: 1. 5-15 g/d; 0. <5/>15g/d. Smoking: 1. never smoker; 0. ever smoker. PA: 1. >=40 min/d walking/bicycling and >=1 h/wk exercise; 0. <40 min/d walking/bicycling and <1 h/wk exercise. BMI: 1. <25 kg/m^2^; 0. >=25 kg/m^2^. | 0 score 1 score 2 scores 3 scores 4 scores 5 scores | Obtained by linkage of the study population with the Swedish National Patient Register and the Swedish Cause of Death Register. | Age, education, aspirin use, history of diabetes, diagnosis of atrial fibrillation, family history of MI before 60 years of age, total energy intake, and Non-Recommended Food Score. |
| Carlsson,A.C. (2013) | The population-based in Stockholm County | Smoking: 1. non-smoker; 0. current smoker. Alcohol drinking: 1. 0.6-30g/d; 0. <0.6/>30g/d. PA: 1. >=1 times/wk; 0. <1 times/wk. Fish: 1. weekly intake; 0. non-weekly intake. Processed meats: 1. <1 times/wk; 0. >= 1 times/wk. Fruit: 1. daily eat; 0. non-daily eat.  Vegetables: 1. daily eat; 0. non-daily eat. | 0-2 scores 3 scores 4-5 scores 6-7 scores | Obtained from the Hospital Care Register and the Cause of Death Register in Sweden. | Educational level and BMI. |
| Ahmed, H. M. (2013) | The Multi-Ethnic  Study of Atherosclerosis | Diet: 1. score above the mediate; 0. score below the mediate. BMI: 1. 18.5 -24.9 kg/m^2^; 0. <18.5 or >24.9 kg/m^2^. Smoking: 1. never smoker; 0. previous/current smoker. PA: 1. >150 min/wk of moderate-intensity activity or >75 min/wk of vigorous intensity activity; 0. <=150 min/wk of moderate-intensity activity or <=75 min/wk of vigorous intensity activity. | 0 score 1 score 2 scores 3 scores 4 scores | Identified  through telephone contact with participants or proxies, death certificates and medical records. | Age, race, gender, study site, and income, baseline CAC, hypertension, hypertension medications, fasting plasma glucose, diabetes medications, non-HDL, HDL-C, TG, lipid-lowering medications, and CRP, baseline CAC. |
| Hoevenaar-Blom, M. P. (2013) | The Monitoring Project on Risk Factors for Chronic Diseases  study | PA: 1. >=3.5 hrs/wk cycling and sports; 0. <3.5 hrs/wk cycling and sports. Diet: 1. Mediterranean Diet Score of 5–8; 0. Mediterranean Diet Score of 0-4. Alcohol drinking: 1. >=1 glass/m; 0. <1 glass/m. Smoking: 1. never or past smoking; 0. currently smoking. Sleep: 1. >=7 hrs/d; 0. < 7 hrs/d. | 0-1score 2 scores 3 scores 4 scores 5 scores | Ascertained through linkage with national registers. Morbidity data were provided by the National Medical Register with the Dutch Hospital Discharge data. Primary and secondary causes of death were obtained through linkage with data from ‘Statistics Netherlands’. | Age, sex, and educational level |
| Wang, Y. (2011) | The population-based health examination surveys | Smoking: 1. never or ever smoking; 0. currently smoking. BMI: 1. < 25 kg/m^2^; 0. >= 25 kg/m^2^. PA: 1. MPA >=150 min/w or VPA >=75 min/w or MVPA >=150 min/w; 0. MPA <150 min/w or VPA <75 min/w or MVPA<150 min/w. Vegetables: 1. >=3 times/wk; 0. <3 times/wk. | 0 score 1 score 2 scores 3 scores 4 scores | Obtained from the Finnish Hospital Discharge Register, the National Social Insurance Institution’s Register, and the Finnish Causes of Death Register. | Age, study year, education, history of MI, history of valvular heart disease, history of diabetes, history of using antihypertensive drugs, SBP, and TC. |
| Zhang, Y. (2011) | The population surveys performed Finland | PA: 1. MPA >=150 min/w or VPA >=75 min/w or MVPA >=150 min/w; 0. MPA <150 min/w or VPA <75 min/w or MVPA<150 min/w. Smoking: 1. never or ever smoking; 0. currently smoking. Alcohol drinking: 1. 1-209g/wk in men, 1-139g/wk in women; 0. none or >=210 g/wk in men, >=140 in women. BMI: 1. < 25 kg/m^2^; 0. >= 25 kg/m^2^. Vegetables: 1. >=3 times/wk; 0. <3 times/wk. | 0-1score 2 scores 3 scores 4 scores 5 scores | Obtained  from Statistics Finland and data on nonfatal events from the National Hospital Discharge Register. | Age, study year, education, family history of stroke, history of diabetes mellitus, SBP, and TC level |
| Ford, E. S. (2009) | The European  Prospective Investigation Into Cancer and Nutrition -Potsdam study | Smoking: 1. never smoking, 0. Smoking. BMI: 1. < 30 kg/m^2^; 0. >= 30 kg/m^2^. PA: 1.>= 3.5 h/wk; 0. < 3.5 h/wk. Diet: 1. above the median of the summed z scores; 0. below the median of the summed z scores. | 0 score 1 score 2 scores 3 scores 4 scores | Identified primarily using self reports, and verified through medical records. | Sex, education, and occupational status. |
| Cardi, M. (2009) | The Seattle Longitudinal Study | Smoking: 1. current smoking; 0. not current smoking. Alcohol drinking: 1. >=14 drinks/wk for men, >=12 drinks/wk for women; 0. <14 drinks/wk for men, <12 drinks/wk for women. Weight: 1. BMI >= 30 kg/m^2^; 0. BMI< 30 kg/m^2^. PA: 1. <3 hr /wk; 0. >=3 hr /wk. Sleep: 1. < 6 hr or > 8 hr a night; 0. 6-8 hr a night. Medical checkups: 1. visit the doctor <1 times/y; 0. visit the doctor >=1 times/y. Dental care: 1. never brushing or flossing one’s teeth and visit the dentist <1 times/y; 0. often brushing or flossing one’s teeth and visit the dentist >=1 times/y. | 0 score 1 score 2 scores 3 scores 4 scores 5 scores | Assessed using medical records available from 1992 to 1998. | NA |
| Lee, C. D. (2009) | The Aerobics  Center Longitudinal Study | Smoking: 1. nonsmoking; 0. smoking. Fitness: 1. physically fit; 0. physically unfit. WC: 1. <94cm; 0.>=94cm. | 0 score 1 score 2 scores 3 scores | Ascertained from responses to mailback health surveys and deaths were identified from the National Death Index and official death certificates. | Age, examination year, alcohol intake, hypertension, diabetes, high cholesterol level, and family history of CHD. |
| Myint, P. K. (2009) | The European Prospective Investigation of Cancer | Smoking: 1. non-smoker; 0. current smoker. PA: 1. >=0.5h/d leisure time activity for sedentary persons or else a non-sedentary occupation with or without leisure time activity; 0. <0.5h/d leisure time activity for sedentary persons. Alcohol drinking: 1. One or more but <14 units/week; 0. none or >14 units/week. Fruit and vegetable: 1. five servings or more as indicated by blood concentration of vitamin C >=50 µmol/l. 0. < five servings as indicated by blood concentration of vitamin C <50 µmol/l. | 0 score 1 score 2 scores 3 scores 4 scores | Ascertained by death certificate data and hospital record linkage. | Age, sex, BMI, SBP, cholesterol concentration, aspirin use, and diabetes mellitus, social class. |
| Kurth, T. (2006) | The prospective  Women’s Health Study | Smoking: 0. Current >=15 cigarettes/d ; 1. Current < 15 cigarettes/d ; 2 .Past >=20 pack-years ; 3. Past < 20 pack-years ; 4. Never. BMI: 0. >=35.0kg/m^2^; 1. 30.0-34.9kg/m^2^; 2. 25.0-29.9 kg/m^2^; 3. 22.0-24.9 kg/m^2^; 4.<22.0 kg/m^2^. PA: 0. Rarely or never ;1. <1 time/wk ; 2. 1 time/wk; 3. 2-3 times/wk; 4. >=4 times/wk . Alcohol drinking: 0. Never; 1. <1 drink/wk; 3. 1-3 drinks/wk ; 4. 4-10.5 drinks/wk ; 2. >=10.5 drinks/wk. Diet : 0. 1st; 1. 2nd; 2. 3rd; 3. 4th; 4. 5th. | 0-4 scores 5-8 scores 9-12 scores 13-16 scores 17-20 scores | Confirmed after review of the medical records by an end-points committee of physicians and cases of fatal stroke were documented using information obtained from death certificates and hospital records. | Age, postmenopausal hormone use, oral contraceptive use, family history of MI, income, geographic location of home, level of education, ethnicity, marital status, and randomized treatment assignments, history of hypertension, antihypertensive treatment, diabetes mellitus, and elevated cholesterol. |

Abbreviations: BMI, body mass index; PA, physical activity; LB, lifestyle behavior; CVD, cardiovascular disease; WC, waist circumference; SBP, systolic blood pressure; DBP, diastolic blood pressure; AHEI, Alternate Healthy Eating Index; US, United States; MI, myocardial infarction; CHD, coronary heart disease; NHS, the Nurses’ Health Study; HPFS, the Health Professionals Follow-up Study; WHR, waist-to-hip ratio; CRP, C-reactive protein; HDL-C, high-density lipoprotein cholesterol; TG, triglyceride; TC, total cholesterol; CAC, coronary artery calcium; MET, metabolic equivalent of task; MPA, moderate physical activity; MVPA, moderate and vigorous physical activity; VPA, vigorous physical activity.

**Supplemental Table 3.** Characteristics of studies related to CVD mortality.

| **Author (year)** | **Cohort** | **Definition of LBs** | **Score category** | **Outcome attainment** | **Adjustments** |
| --- | --- | --- | --- | --- | --- |
| Troeschel, A. N. (2023) | The Prospective REasons in Stroke Study | Alcohol drinking: 0. 0drinks/d; 1. >0–<=1 drink/d for women and >0–<=2 drinks/d for men; 2. >1 drink/d for women and >2 drinks/d for men. PA: 0. 0 times/week; 1. 1–3 times/week; 2. >=4 times/week. BMI: 0. <25.0 kg/m^2^;1. 25–<30 kg/m^2^; 2. >=30 kg/m^2^. Smoking: 0. not current;1. current. | Q1 Q2 Q3 Q4 Q5 | Identified through telephone contact with participants or proxies, adjudicated by medical records, death certificates, and a committee of trained adjudicators. | Age, sex/hormone replacement therapy use, race, income, education, insurance, marital status, region, co-morbidities, aspirin/ non-steroidal anti-  inflammatory drug use, statin use, total energy intake, former smoking status and the dietary inflammation score. |
| Wang, T. (2022) | The follow-up Study in Jiangxi Province of China | Alcohol drinking: 1. <25g of pure alcohol in men and <15g in women per day; 0. >=25g of pure alcohol in men and >=15g in women per day. Smoking: 1. never smoking or past smoking; 0. current smoking. Diet(AHEI scores): 1. highest 40% AHEI-2010 score; 0. lower 60% AHEI-2010 score. PA: 1. 3 days of vigorous activity of at least 20min/d or 5 days of moderate intensity activity or walking of >30min/d for >10minutes at a time or achieving at least 600 MET-min/wk; 0. 3 days of vigorous activity of < 20min/d or 5 days of moderate intensity activity or walking of <=30min/d for <=10minutes at a time or achieving < 600 MET-min/wk. | 0-2 scores 3 scores 4 scores | Identified through the Jiangxi Province Disease Surveillance scores system, telephone contact with DSP death registries, local residential records, home phone answering and township hospital, adjudicated by medical records, death certificates. | Age, gender, BMI, WC, hypertension, anti-hypertensive drugs, and stroke. |
| Heath, L. (2022) | The UK Biobank | Smoking: 1. never smoker; 0. smoker. Alcohol drinking: 1. < 112g/wk; 0. >=112 g/wk. Diet: 1. >=5 servings of fruit and vegetable/d; 0. < 5 servings of fruit and vegetable/d. PA: 1. moderate-vigorous activity >=150 min/wk; 0. moderate-vigorous activity <150 min/wk. | 0 score 1 score 2 scores 3 scores 4 scores | Identified by Hospital admission data and Death registries. | Age, sex, ethnicity, Townsend Deprivation Score, education, region, family history of CVD, family history of diabetes, menopausal status, and BMI group. |
| Hu, P. (2022) | The prospective Guangzhou Heart Study | BMI:1. 18.5–23.9 kg/m^2^; 0. <18.5 kg/m^2^ and >23.9 kg/m^2^. Alcohol drinking: 1. never drinking or alcohol cessation or occasional drinking; 0. frequent drinking. Smoking:1. have never smoked or have smoked< 100 cigarette; 0. have currently smoked or smoked >=100 cigarettes. Diet quality: 1. healthy diet; 0. unhealthy diet. LTPA: 1. reach the minimum level of the recommended standard by WHO; 0. not reach the minimum level of the recommended standard by WHO. Sleep quality: 1. good sleep quality; 0. poor sleep quality. Mental status: 1. unhealthy mental status; 0. healthy mental status. | 0-2 points 3-5 points 6-7 points | Identified by China’s National Death Registry, Guangzhou Center for Disease Control and Prevention. | Age, sex, marital status, educational status. |
| Kim, S. (2022) | The Korean longitudinal study of aging | Smoking:1. currently or past smoker; 0. never smoker. Alcohol drinking:1. > 5drinks/w; 0. <5 drinks/w Weight: 1. <18.5 or BMI > 25 kg/m^2^; 0. >=18.5 and <=25 kg/m^2^. PA: 1. <2 times/w; 0. >=2times/w. unintentional weight loss: 1. weight loss of >=5Kg; 0. weight loss of <5Kg. | 0 point 1 point 2 points 3-5 points | Crosschecked by the acquired information to the death records from the national statistical office in Korea. | Age, household income, education, occupation, religion, type of housing, cognition, depressive symptoms, comorbidity, medications, activities of daily living condition, fall experience, and hospitalization. |
| Ibsen, D. B. (2021) | The Danish Diet, Cancer and Health cohort | Smoking: 1. never smoker or ever smoker; 0. current smoker. Alcohol drinking: 1. <=7 units /wk for women and <=14 units/wk for men; 0. >7 units /wk for women and >14 units/wk for men. Diet: 1. <3 scores (low adherence); 0.>= 5 scores (high adherence). PA: 1. >=30 min/d of moderate-to-vigorous activity; 0. <30 min/d of moderate-to-vigorous activity. | 0 point 1 point 2 points 3 points 4 points | Identified through linkage to The Danish Civil Registration System, and the Danish Register of Causes of Death. | Age, date at study entry, sex, education, BMI, diet score, smoking status, alcohol consumption, and PA. |
| Li, Z. (2021) | The prospective Iowa Women’s Health Study | Alcohol drinking: 2. > 7drinks/w; 1. >0 to <=7drinks/w; 0. none. PA: 2. low activity; 1. vigorous activity 1time/w and moderate activity 1time/w or moderate activity 2–4 times/w; 0. vigorous activity 2 times/w or moderate activity >4 times/w. Smoking: 1. currently smoker; 0. former and never smoker. BMI: 2. >=30 kg/m^2^; 1. 25–29.99 kg/m^2^; 0. <25 kg/m^2^. | Q1 Q2 Q3 Q4 Q5 | Identified by the State Health Registry of Iowa and the National Death Index. | Age, total energy intake, education, marital status, comorbidity score, hormone replacement therapy use, former smoker, and unweighted dietary inflammation score. |
| Liu, G. (2021) | The Chinese Longitudinal Healthy longevity Survey project | Diet: 1. >=4 scores on a modified version of the Mediterranean diet score; 0. <4 scores on a modified version of the Mediterranean diet score. Smoking: 1. never smoking; 0. ever or current smoking. Alcohol drinking: 1. <14U/wk; 0. >=14U/wk. PA: 1. >=150 minutes of moderate-to-vigorous intensity activity per week; 0. <150 minutes of moderate-to-vigorous intensity activity per week. | 0 point 1 point 2 points 3-4 points | NA | Sex, age, marital status, educational background, residence, economic income and BMI |
| Sotos-Prieto, M. (2021) | The Study on Nutrition and Cardiovascular Risk in Spain | Smoking: 1.not smoking; 0. Smoking.  BMI: 1. <25 kg/m2; 0. >=25 kg/m2. PA: 1. >=2.5h/wk; 0. 2.5h/wK. TV: 1. <=7 h/wk; 0. >7 h/wk. Diet: 1. top 40% of the AHEI -2010; 0. bottom 60%of the Alternative Healthy Eating Index-2010.  Alcohol drinking: 1. 0.1–14.9g/d; 0. <0.1 or >14.9g/d. | Q1 Q2 Q3 Q4 | Identified by the Spanish National Institute of Statistics and death certificate. | Sex, age, educational level, smoking, total energy intake, and BMI, prevalence of cancer, respiratory disease, depression, number of morbidities, number of drug treatments, abdominal obesity, FBG, high blood pressure or receiving antihypertensive drugs, TG>= 150mg/dl and serum HDL-C < 40mg/dl in men or < 50mg/dl in women. |
| Troeschel, A. N. (2021) | The REasons for Geographic and Racial Differences in Stroke study | Diet: 1. score 17-33; 2. score 34-37; 3. score 38-40; 4. score 41-44; 5. score 45-60. PA: 1. 0 times/wk; 2. 1-2 times/wk; 3. 3-4 times/wk; 4. 5-6 times/wk; 5. >=7 times/wk. Social network size: 1. 0-5; 2. 6-7; 3. 8-11; 4. 12-17; 5. >=18. Alcohol drinking: 1. >=7.5drinks/wk for men or >=6.0 drinks/wk for women; 2. 3.5-7.0 drinks/wk for men or 2.0-5.0 drinks/wk for women; 3. 1.0-3.0 drinks/wk for men or 0.5-1.75 drinks/wk for women; 4. >0.0-<1.0 drinks/wk for men or >0.0-<0.5 drinks/wk for women; 5. 0 drinks/wk. Smoking: 1. >=37.1pack/yrs; 2. 18.0-37.0 pack/yrs; 3. 5.1-17.9 pack/yrs; 4. <5.1 pack/yrs; 5. 0 pack/yrs. WC: 1. >=109.3cm for men and >=104.2cm for women; 2. 101.1-109.2cm for men and 94.1-104.1cm for women; 3. 96.0-101.0cm for men and 86.5-94.0cm for women; 4. 89.0-95.9cm for men and 78.2-86.4cm for women; 5. 61.0-88.9cm for men and 55.9-78.1cm for women. Sedentary: 1. >=4hrs/d; 2. 3hrs/d; 3. 2 hrs/d; 4. 1 hrs/d; 5. <1 hrs/d. | Q1 Q2 Q3 Q4 Q5 | Identified through telephone contact with participants or proxies, adjudicated by medical records, death certificates, and a committee of physicians. | Age, gender, BMI, WC, hypertension, anti-hypertensive drugs, and stroke. |
| Zhang, X. (2021) | The China Patient-centered Evaluative Assessment of Cardiac Events Million Persons Project | Smoking: 1. never smokers or former smokers who stopped by choice as recommended; 0. current smokers. Alcohol drinking: 1. never drinkers, or <= 25g (for male) or 15g (for female) per day; 0. >25g (for male) or 15g (for female) per day. LTPA: 1. >= 150min/wk of moderate-intensity aerobic activities or 75min/wk of vigorous -intensity aerobic activities; 0. <150min/wk of moderate-intensity aerobic activities or 75min/wk of vigorous -intensity aerobic activities. Diet: 1. score>=4; 0. score<4. | 0 score 1 score 2 scores 3 scores 4 scores | Ascertained through the National Mortality Surveillance System and Vital Registration of Chinese Center for Disease Control and Prevention. | Age, sex, occupation, education, household income, marriage, social medical insurance, urbanicity, region, and county level per capital Gross Domestic Product. |
| Lee, D. H. (2020) | The Korea National Health and Nutrition Examination Survey | BMI: 1. <18.5 or >=25 kg/m^2^; 0. 18.5-25 kg/m^2^. Smoking: 1. current smoking; 0. not current smoking. Drinking: 1. >=14 drinks/wk for men and >=10 drinks/wk for women; 0. <14 drinks/wk for men and <10 drinks/wk for women. PA: 1. >=150 min/wk of moderate-intensity activity, >=75 min/wk of vigorous-intensity activity, or a combination of them; 0. <150 min/wk of moderate-intensity activity or <75 min/wk of vigorous-intensity activity. Sleep: 1. <7 or >=9 hr/d; 0. 7-9hr/d. | 0 score 1 score 2 scores 3 scores 4-5 scores | Identified by death certificates and medical records. | Sex, age, education, income, occupation, regional area, and marital status. |
| Wu, M. Y. (2020) | A prospective cohort based on the Yinzhou Health Information System in China | BMI: 0. 18.5-<24kg/m^2^; 1. <18.5 or >=24 kg/m^2^. Smoking: 0. Never smoking; 1. Current or former smoking. Alcohol drinking: 0. never drinking; 1. often and occasionally drinking. PA: 0. aerobic exercise >=80 min/wk; 1. aerobic exercise <80 min/wk. | Q1 Q2 Q3 Q4 | Identified by Yinzhou Death Database and Yinzhou Centers for Disease Control and Prevention. | Age, sex, marital status, and educational attainment, and 4 lifestyle-related factors (BMI level, smoking, alcohol drinking, and PA). |
| Bonaccio, M. (2019) | The Moli-sani Study | Smoking: 1. ever and former smokers; 0. current smokers. Diet: 1. above each sub-population-specific median; 0. below each sub-population-specific median. PA: 1. >=30 min/d; 0. <30 min/d. Obesity: 1. WHR<0.85 for women or <0.90 for men; 0.WHR>=0.85 for women or >=0.90 for men. | 0-1 score 2 scores 3 scores 4 scores | Identified by follow-up. Information on lifestyles was collected by in-person interviews, anthropometric measures and by trained personnel. Dietary information was obtained through the Italian EPIC food frequency questionnaire. | Age, sex, educational level, household income, energy intake, treatment for diabetes, medication for hypertension, lipid-lowering drugs, CVD, cancer, use of antiplatelet drugs. |
| Zhu, N. (2019) | The China Kadoorie Biobank Study | Smoking: 1. never smoking or smoking cessation not due to illness; 0.current smoking. PA: 1. above sex-specific median of total physical activity level; 0. below sex-specific median of total physical activity level. BMI: 1.18.5 - 27.9 kg/m2; 0. <18.5 or >27.9 kg/m2. Alcohol drinking: < 30 g/d in men and < 15 g/d in women; 0. >=30 g/d in men and >=15 g/d in women. Diet: 1. 4-5scores; 0. <4 scores. WC (M/F): 1. < 90 cm in men and < 85 cm in women; 0. >=90 cm in men and >= 85 cm in women. | 0 score 1 score 2 scores 3 scores 4 scores 5 scores | Identified through Chinese Disease Surveillance scores system, linkages with DSP death registries and local residential records, combined with annual active follow-up. | Sex, education, marital status, family histories of heart attack, stroke or cancer, and hip circumference. |
| Han, C. (2018) | The China-PAR project | Diet: 1. score >=2 components; 0. score <2 components. Smoking: 1. never or former smoker; 0. current smoker. BMI: 1. <25 kg/m^2^; 0. >= 25kg/m^2^. PA: 1. >=30 min/d of moderate-to-vigorous activity; 0. <30 min/d of moderate-to-vigorous activity. | 0-1 score 2 scores 3 scores 4 scores | Collected through hospital records or death certificates. | Age, sex, living region, urbanization, drinking status, education level, family history of atherosclerotic cardiovascular disease, and cohort sources. |
| Li, Y. (2018) | The Nurses’ Health Study & the Health Professionals Follow-up Study | Smoking: 1. never smoking; 0. ever or current smoking. BMI: 1. 18.5-24.9 kg/m^2^; 0. <18.5 or 24.9 kg/m^2^. PA: 1. >=30 min/d of moderate to vigorous activity; 0. < 30 min/d of moderate to vigorous activity. Alcohol drinking: 1. 5-15 g/d in women or 5-30 g/d in men. Diet: 1. upper 40% of AHEI; 0. below 40% of AHEI. | 0 score 1 score 2 scores 3 scores 4 scores 5 scores | Identified from the NHS and HPFS, state vital statistics records, the National Death Index, reports by the families, and the postal system. | Age; sex; ethnicity; current multivitamin use; current aspirin use; family history of diabetes mellitus, MI, or cancer; and menopausal status and hormone use (women only). |
| Zhang, Q. L. (2017) | The Shanghai Men’s Health Study (2002–2013) | Smoking: 1. current smoker or quitting smoking<10 years; 0. never smoker or quitting smoking>=10 years. Alcohol drinking: 1. >14 drink/wk; 0. < 14 drinks/wk. Diet: 1. Chinese food pagoda score distributed in the bottom two quintile in the SMHS; 0. Chinese food pagoda score distributed in the top three quintile in the SMHS. PA: 1. <2 MET-hrs/d moderate-to-vigorous-intensity activity; 0. >=2 MET-hrs/d moderate-to-vigorous-intensity activity. | 0 score 1 score 2 scores 3 scores 4 scores | Identified through annual record linkages to the Shanghai Cancer Registry and the Shanghai Vital Statistics. | Age, education, income per person, occupation, history of hypertension, diabetes mellitus, CHD, and stroke. |
| Fazel-Tabar Malekshah, A. (2016) | The Golestan Cohort Study | BMI: 0. >=25kg/m^2^; 1. 18.5–24.9kg/m^2^. Diet: 0. score in lower three fifth; 1. score in upper two fifth. PA:0. < 30 min/d; 1. >=30 min/d. | 0 score 1 score 2 scores 3 scores | Identified through telephone contact with participants or friends and local health workers of participants, adjudicated through validated verbal autopsy questionnaires by interviewing the closest relative of the dead person | Age, gender, resident area, ethnicity, socioeconomic status, marital status, education, alcohol consumption, opium use, BMI. |
| Lohse, T. (2016) | The MONItoring of trends and determinants in CArdiovascular disease and the National Research Program 1A, a community based primary prevention of CVD | BMI: 1. 18.5-24.9 kg/m^2^; 0.5. 25–29.9 kg/m^2^; 0. <18.5 or >=30 kg/m^2^. PA: 1. >=2 d/wk; 0.5. 1 d/wk; 0. <1 d/wk. Sedentary behavior: 0. mostly sitting; 0.5. walking, cycling, other regular activities such as gardening; 1. regular exercise. Energy density: 0. score 0; 0.5. score 1; 1. score 2 or 3. Fruits and vegetables: 0. no fruits or vegetables; 0.5. either fruits or vegetables; 1. both fruits and vegetables. Grains: 0. Not eat relatively unprocessed cereals (grains) and/or pulses (legumes) with every meal; 1. Eat relatively unprocessed cereals (grains) and/or pulses (legumes) with every meal. Alcoohol drinking: 0. >=2 drinks/d for men and 1 drink/d for women; 1. <2 drinks/d for men and 1 drink/d for women. Processed meat: 0. sausage products; 0.5. meat; 1. none. Salt: 0. always eat salt-preserved, salted, or salty foods; 0.5. sometimes eat salt-preserved, salted, or salty foods; 1. never eat salt-preserved, salted, or salty foods. | 1 score 2 scores 3 scores | Identified by census and death registry–linked survey data. | Education, marital status, study, language region, nationality, and smoking status. |
| Warren Andersen, S. (2016 AF) | The Southern Community Cohort Study | Smoking: 1. never smoker; 0. former or current smoker. Alcohol drinking: 1. 0-1drinks/d for women and 0-2drinks/d for men; 0. >1drinks/d for women and >2drinks/d for men. PA: 1. >=150 min/wk of moderate-intensity, or >=75 min/wk of vigorous-intensity activity, or >=150 min/wk of combination of them; 0. <150 min/wk of moderate intensity, or< 75 min/wk of vigorous-intensity activity, or < 150 min/wk of combination of them. Sedentary behavior: 1. <=5.75 hrs/d; 0. >5.75 hrs/d. | 0 score 1 score 2 scores 3 scores >=4 scores | Identified from the National Death Index though December 31, 2011 (data analyzed in 2014–2015). | Enrollment source, education, income, marital status, neighborhood deprivation, and BMI. |
| Warren Andersen, S. (2016 WH) | The Southern Community Cohort Study | Smoking: 1. never smoker; 0. former or current smoker. Alcohol drinking: 1. 0-1drinks/d for women and 0-2drinks/d for men; 0. >1drinks/d for women and >2drinks/d for men. PA: 1. >=150 min/wk of moderate-intensity, or >=75 min/wk of vigorous-intensity activity, or >=150 min/wk of combination of them; 0. <150 min/wk of moderate intensity, or< 75 min/wk of vigorous-intensity activity, or < 150 min/wk of combination of them. Sedentary behavior: 1. <=5.75 hrs/d; 0. >5.75 hrs/d. | 0 score 1 score 2 scores 3 scores >=4 scores | Identified from the National Death Index though December 31, 2011 (data analyzed in 2014–2015). | Enrollment source, education, income, marital status, neighborhood deprivation, and BMI. |
| Taheri, Shahrad (2015 EU) | The tri-ethnic cohort study, the Southall and Brent Revisited | Smoking: 1. not currently smoking; 0. currently smoking. Alcohol drinking: 1. 1- 14 units/wk for women and 1- 21 units/wk for men; 0. <1 or >14 units/wk for women and < 1or >21 units/wk for men. PA: 1. >=5 times/wk moderate exercise or >=2.5 times/wk vigorous exercise; 0. <5 times/wk moderate exercise or <2.5 times/wk vigorous exercise. Fruit and vegetables: 1.>= 5.5 times/wk of either fruit, or vegetables, or both; 0. <5.5 times/wk of either fruit, or vegetables. | 0 score 1 score 2 scores 3 scores 4 scores | Identified through National Statistics. | Age, sex, BMI, DBP, SBP, hypertension treatment, TC, HDL-C, social class, employment, and occupational PA. |
| Taheri, Shahrad (2015 SA) | The tri-ethnic cohort study, the Southall and Brent Revisited | Smoking: 1. not currently smoking; 0. currently smoking. Alcohol drinking: 1. 1- 14 units/wk for women and 1- 21 units/wk for men; 0. <1 or >14 units/wk for women and < 1 or >21 units/wk for men. PA: 1. >=5 times/wk moderate exercise or >=2.5 times/wk vigorous exercise; 0. <5 times/wk moderate exercise or <2.5 times/wk vigorous exercise. Fruit and vegetables: 1.>= 5.5 times/wk of either fruit, or vegetables, or both; 0. <5.5 times/wk of either fruit, or vegetables. | 0 score 1 score 2 scores 3 scores 4 scores | Identified through National Statistics. | Age, sex, BMI, DBP, SBP, hypertension treatment, TC, HDL-C, social class, employment, and occupational PA. |
| Hoevenaar-Blom, M. P. (2014) | The Monitoring Project on Risk Factors for Chronic Diseases | PA: 1. >=3.5 h/wk cycling or sports; 0. <3.5 h/wk cycling or sports. Diet: 1. Mediterranean Diet Score 5-8; 0. Mediterranean Diet Score 0-4. Alcohol drinking: 1.>=1 beverage/month; 0. < 1 beverage/month. Smoking: 1. non-smoking; 0. smoking. Sleep: 1. >=7 hrs/d; 0. <7 hrs/d. | 0-1 score 2 scores 3 scores 4-5 scores | Provided by the National Medical Registerwith the Dutch Hospital Discharge data and primary and secondary causes of death were obtained through linkage with data from ‘Statistics Netherlands’. | Age, sex, and educational level. |
| Eguchi, E. (2012) | The Japan collaborative cohort study | Fruits: 1. >=1 intake/d; 0. <1 intake/d. Fish: 1. >=1 intake/d; 0. <1 intake/d. Milk: 1. almost every day; 0. not every day. PA: 1. >=5 h/wk and/or walking >=1 h/d; 0. <5 h/wk and/or walking<1 h/d. BMI: 1. 21–25 kg/m2; 0. <21 or >25 kg/m2. Alcohol drinking: 1. <=46.0g/d; 0. >46.0g/d. Smoking: 1. never or ever smoker; 0. current smoker.  Sleep: 1. 5.5–7.5 h/d; 0. <5.5 or >7.5 h/d. | 0-2 scores 3 scores 4 scores 5 scores 6 scores 7-8 scores | Identified by reviewing all death certificates in each area. | Age, history of hypertension, history of diabetes, education level, regular employment, and perceived mental stress. |
| Ford, E. S. (2011) | The National Health and Nutrition Examination Survey III Mortality Study | Smoking: 1. never smoker; 0. ever or current smoker. Diet: 1. top 40% of the Healthy Eating Index; 0. bottom 60% of the Healthy Eating Index. PA: 1. >=3 times/wk activity; 0. < 3 times/wk activity. Alcohol drinking: 1. >0-<=60 drinks/m for men or >0-<=30 drinks/m for women; 0. >60 drinks/m for men or >30 drinks/m for women. | 0 score 1 score 2 scores 3 scores 4 scores | Ascertained with the National Death Index. | Age, gender, race or ethnicity, and educational status. |
| McCullough, M. L. (2011) | The Cancer Prevention Study-II Nutrition Cohort | BMI: 0. >=30 kg/m^2^ at 1 or both time scores; 1. other combinations; 2. 18.5–24.9 kg/m^2^ in 1982 and 1992. PA: 0. <8.75 MET-h/wk; 1. 8.75-<17.5 MET-h/wk; 2. >=17.5 MET-h/wk. Alcohol drinking: 0. >2 drinks/d; 1. Nondrinker; 2. Drinker of <=2 drinks/d. Diet: 0. score<3; 1. score 3-<6; 2. score >=6. | 0-2 scores 3 scores 4 scores 5 scores 6 scores 7-8 scores | Determined through December 31, 2006 by linkage with the National Death Index | Age, smoking status, and education. |
| Odegaard, A. O. (2011) | The Singapore Chinese Health Study | Diet: 0. Lowest 40% of dietary pattern score; 1. Upper 60% of dietary pattern score. PA: 0. <2 h/wk of moderate or no strenuous activity; 1. >=2 h/wk of moderate or any strenuous activity. Alcohol drinking: 0. None or >2 drinks/d; 1. 1-14 drinks/wk. Sleep: 0. <6 or >=9h/d; 1. 6-8h/d. Smoking: 0. Ever smoked; 1. Never smoked. BMI: 0. <18.5 and >21.5 kg/m2(Age<65 y) or <18.5 and >24.5 kg/m2(Age>=65 y); 1. 18.5-21.5 kg/m2(Age<65 y) or 18.5-24.5 kg/m2(Age>=65 y). | 0-1 score 2 scores 3 scores 4 scores 5-6 scores | Obtained through linkage analysis with the nationwide registry of birth and death in Singapore. | Age, sex, year of enrollment, dialect, education, marital status, and energy intake. |
| Kvaavik, E. (2010) | The Health and Lifestyle Survey | Smoking: 1. current smoker; 0. ever or never smoker.  Fruits and vegetables: 1. < 3 times/d; 0. >=3 times/d. PA: 1. <=2 hrs/wk; 0. >2 hrs/wk. Alcohol drinking: 1. >14 U/wk in women and > 21U/wk in men; 0. <=14 U/wk in women and <= 21U/wk in men. | 0 score 1 score 2 scores 3 scores 4 scores | Obtained from the United Kingdom National Health Service Central Registry, and deaths from all causes, CVD, cancer and other causes were ascertained from death certificates. | Age, sex, occupational social class, BMI, blood pressure, and prior diagnosis of heart disease, angina, heart attack, high blood pressure, stroke, arterial disease and cancer, diabetes mellitus, bronchitis, emphysema, asthma, respiratory tuberculosis, and other respiratory tract diseases. |
| Mitchell, J. A. (2010) | The Aerobics Center Longitudinal Study | CRF: 1. high CRF (the upper two thirds); 0. low CRF (the lowest third). PA: 1. moderate (walk, jog, or run up to 10 miles/wk or join in sporting or leisure-time physical activity other than walking, jogging, or running) or high (walk, jog, or run >10 miles /wk); 0. no activity. Smoking: 1. ever pr never smoker; 0. current smoker.  Alcohol drinking: 1. 1-14 drinks/wk; 0. 0 or >14 drinks/wk. BMI: 1. 18.5-24.9 kg/m^2^; 0. >=25.0 kg/m^2^. | 0 score 1 score 2 scores 3 scores 4 scores 5 scores | Determined from the National Death Index report or by review of death certificates, the latter being obtained from the department of vital records in the decedent's state of residence. | Age, examination year, hypertension, diabetes, hypercholesterolemia, low HDL-C, and family history of CVD. |
| Nechuta, S. J. (2010) | The Shanghai Women’s Health Study | BMI: 0. <18.5 or >24.99 kg/m^2^; 1. 18.5-24.99 kg/m^2^. WHR: 0. >=0.786; 1. <0.786. PA: 0. <2 MET h/d; 1. >=2 MET h/d. Spouse smoke: 0. Ever exposed to spouse’s smoking; 1. Never exposed to spouse’s smoking. Fruit and vegetable: 0. <626.5 g/d; 1. >=626.5 g/d. | 0 score 1 score 2 scores 3 scores 4-5 scores | Obtained by annual linkage to the population-based Shanghai cancer and vital statistics registries. | Age, education, occupation, and income. |
| Lee, C. D. (2009) | The Aerobics Center Longitudinal Study | Smoking: 1. nonsmoking; 0. smoking. Fitness: 1. physically fit; 0. physically unfit. WC: 1. <94cm; 0.>=94cm. | 0 score 1 score 2 scores 3 scores | Ascertained from responses to mail-back health surveys. | Age, examination year, alcohol intake, hypertension, diabetes, high cholesterol level, and family history of CHD. |
| Khaw, K. T. (2008) | The European Prospective Investigation into Cancer and Nutrition. | Smoking: 1. current non-smoking; 0. current smoking. Alcohol drinking: 1. 1-14U/wk; 0. 0 or >14U/wk. PA: 1. sedentary job with >=0.5 h/d recreational activity or standing job; 0. sedentary job with no recreational activity. Fruit and vegetable intake: indicated by Blood Vitamin C level, 1. >=50 mmol/l; 0. <50 mmol/l. | 0 score 1 score 2 scores 3 scores 4 scores | Identified by death certification at the Office of National Statistics, United Kingdom. | Age, Sex, and BMI. |
| Knoops, K. T. (2004) | The Healthy Ageing: a Longitudinal study in Europe project | Diet：1. >=4 scores on a modified version of the Mediterranean diet score; 0. <4 scores on a modified version of the Mediterranean diet score. Alcohol drinking: 1. >0g/d; 0. 0g/d. PA: 1. score in the intermediate and the highest tertile; 0. score in the highest tertile. Smoking: 1. never smoking or had stopped smoking >15 years; 0. current smoking or had stopped smoking <15 years. | 0-1 score 2 scores 3 scores 4 scores | Collected every 5 years in FINE and in 1999-2000 for the Survey in Europe on  Nutrition and the Elderly: a Concerned Action and was available for 99.7% of participants. | Sex, age, BMI, and study population. |
| Luoto, R. (1998) | Follow-up study towards persons who had answered the questionnaire that was sent by the Finnish National Public Health Institute | Smoking: 0. non-smoker; 1. smoker. PA: 0. >=2 times/m; 1. <2 times/m. Diet: 1. top 40% of the Alternative Healthy Eating Index-2010; 0. bottom 60%of the Alternative Dairy fat: 0. not use of butter on bread and use of whole milk containing <3.9% fat; 1. use of butter on bread and use of whole milk containing >= 3.9% fat. | 0 score 1 score 2 scores 3 scores | Linked with mortality statistics from 1978 to 1993 from the Central Population Register by means of the unique personal identification numbers for all residents of Finland since January 1, 1967. | Age, chronic morbidity, education, BMI, and period. |

Abbreviations: BMI, body mass index; PA, physical activity; LB, lifestyle behavior; WC, waist circumference; AHEI, Alternate Healthy Eating Index. UK, United Kingdom; MET, metabolic equivalent of task; CVD, cardiovascular disease; HDL-C, high density lipoprotein cholesterol; TG, triglyceride; FBG, fasting blood glucose; MI, myocardial infarction; MET, metabolic equivalent of task; CHD, coronary heart disease; DBP, diastolic blood pressure; SBP, systolic blood pressure; CRF, cardiorespiratory fitness; WHR, waist-to-hip ratio; AF, African American; WH, White; EU, Europeans; SA, South Asians; LTPA, leisure-time physical activity; WHO, World Health Organization; M, male; F, female; NA, not reported.

**Supplemental Table 4.** Characteristics of studies related to CVD recurrence, mortality and all-cause mortality among individuals with CVD.

| **Author (year)** | **Cohort** | **Definition of LBs** | **Score category** | **Outcome attainment** | **Adjustments** |
| --- | --- | --- | --- | --- | --- |
| Yang, Y. L. (2021) | The Biosignature coronary artery disease study | BMI: 1. 20–24.99 kg/m^2^; 0. <20 or >24.99 kg/m^2^. Diet: 1. >= 5 points (high adherence); 0. <3 points (low adherence). Smoking: 1. had never smoked or had quit smoking >6 months; 0. current smoking or had quit smoking <=6 months. PA: 1. exercise >= 30 minutes and >= five times a week; 0. exercise < 30 minutes and < five times a week. | 1 score 2 scores 3 scores 4 scores | Identified and recorded during follow-up. | Age, gender, history of hypertension, diabetes, and medications. |
| Booth, J. N. (2014) | The REasons for Geographic and Racial Differences in Stroke study | PA: 1. >=4 times/wk; 0. < 4 times/wk. Smoking: 1. nonsmoking; 0. smoking. Diet: 1. highest quartile of Mediterranean diet score; 0. lowest three fourths of Mediterranean diet score. WC: 1. <88 cm for women and <102 cm for men; 0. >=88 cm for women and >=102 cm for men. | 0 score 1 score 2 scores 3-4 scores | Collected through a telephone interview, self-administered questionnaires, and an inhome examination. | Age, race, sex, region of residence, education and income, LDL-C, SBP, DBP, WC, self-rated health, diabetes, albuminuria, estimated glomerular filtration rate, C-reactive protein, aspirin use, clopidogrel use, beta blocker use, angiotensin converting enzyme inhibitor use, angiotensin receptor blocker use, and statin use. |
| Towfighi, A. (2012) | The National Health and Nutrition Examination Survey | Diet: 1. >=5 servings of fruits/vegetables per day; 0. <5 servings of fruits/vegetables per day.  PA: 1. >12 times/m; 0. <=12 times/m.  BMI: 1. 18.5-29.9 kg/m2; 0. <18.5 or >29.9 kg/m^2^. Alcohol drinking: 1. 1 drink/day for women and 2 drinks/day for men; 0. not 1 drink/d for women and 2 drinks/d for men. Smoking: 1. not smoking; 0. smoking. | 0 score 1 score 2 scores 3 scores 4 scores 5 scores | Recorded from NHANES III mortality follow-up data, which relied on a probabilistic match between NHANES III and National Death Index death certificate records. | Age, sex, race, hypercholesterolemia, hypertriglyceridemia, diabetes mellitus, low HDL-C, hypertension, and history of MI. |
| Han, Y. (2021) | The China Kadoorie Biobank study | Smoking: 1. current smokers and former smokers who quit because of illness to the high-risk group; 0. not current smokers.  Alcohol drinking: 1.>=30 g/d or having stopped drinking; 0. 0-30 g/d. Diet: 1. non-daily eating of vegetables, fruits, and eggs, and eating red meat daily or less than weekly. 0. daily eating of vegetables, fruits, and eggs, and eating red meat daily or more than weekly.  PA: 1. below the mediate; 0. above the mediate. Body shape: 1. BMI<18.5 or >=28.0 kg/m2 or having WC >=90 cm (men)/85 cm (women); 0. BMI 18.5-28.0kg/m2 or having WC<90 cm (men)/85 cm (women). | 0-1 score 2 scores 3 scores 4 scores 5 scores | Identified through linkages to disease and mortality registries and national health insurance claim database, supplemented with local residential records and annual active confirmation. | Sex, education, marital status, parental family history of cardiometabolic multimorbidity. |

Abbreviations: BMI, body mass index; PA, physical activity; LB, lifestyle behavior; WC, waist circumference; CHD, coronary heart disease; SBP, systolic blood pressure; DBP, diastolic blood pressure; WC, waist circumference; LDL-C, Low density lipoprotein cholesterol; NHANES, The National Health and Nutrition Examination Survey; MI, myocardial infarction; HDL-C, high density lipoprotein cholesterol.

**Supplemental Table 5.** Assessment of quality of included studies (Newcastle-Ottawa Quality Assessment Scale).

| **Study** | **Term1** | **Term2** | **Term3** | **Term4** | **Term5** | **Term6** | **Term7** | **Term8** | **Term9** | **Total** |
| --- | --- | --- | --- | --- | --- | --- | --- | --- | --- | --- |
| Mo, M.(2023) | 1 | 1 | 0 | 1 | 1 | 1 | 1 | 1 | 1 | 8 |
| Mao, Ziling(2023) | 0 | 1 | 1 | 1 | 1 | 1 | 1 | 1 | 0 | 7 |
| Zuo, Y.(2022) | 1 | 1 | 1 | 1 | 1 | 1 | 1 | 1 | 0 | 8 |
| Heath, L.(2022) | 1 | 1 | 1 | 1 | 1 | 1 | 1 | 1 | 0 | 8 |
| Guasch-Ferré, Marta(2022 NHS) | 0 | 1 | 1 | 1 | 1 | 1 | 1 | 1 | 0 | 7 |
| Guasch-Ferré, Marta(2022 HPFS) | 0 | 1 | 1 | 1 | 1 | 1 | 1 | 1 | 0 | 7 |
| Yang, R.(2021) | 1 | 1 | 1 | 1 | 1 | 1 | 1 | 1 | 1 | 9 |
| Han, Y.(2021) | 1 | 1 | 1 | 1 | 1 | 1 | 1 | 1 | 0 | 8 |
| Tsai, Ming-Chieh(2021) | 1 | 1 | 0 | 1 | 1 | 1 | 1 | 1 | 0 | 7 |
| Dimovski, K.(2019) | 1 | 1 | 0 | 1 | 1 | 1 | 1 | 1 | 0 | 7 |
| Diaz-Gutierrez, J.(2018) | 1 | 1 | 1 | 1 | 1 | 1 | 1 | 1 | 1 | 9 |
| Lv, J.(2017) | 1 | 1 | 1 | 1 | 1 | 1 | 1 | 0 | 1 | 8 |
| Larsson, S. C.(2016 CSM) | 1 | 1 | 0 | 1 | 1 | 1 | 1 | 1 | 0 | 7 |
| Larsson, S. C.(2016 SMC) | 1 | 1 | 0 | 1 | 1 | 1 | 1 | 1 | 0 | 7 |
| Chomistek, A. K.(2015) | 0 | 1 | 0 | 1 | 1 | 1 | 1 | 1 | 0 | 6 |
| Del Gobbo, L. C.(2015) | 0 | 1 | 1 | 1 | 1 | 1 | 1 | 1 | 0 | 7 |
| Akesson, A.(2014) | 0 | 1 | 1 | 1 | 1 | 1 | 1 | 1 | 0 | 7 |
| Agha, G.(2014) | 0 | 1 | 0 | 1 | 1 | 1 | 1 | 1 | 0 | 6 |
| Larsson, S. C.(2014) | 0 | 1 | 1 | 1 | 1 | 1 | 1 | 1 | 0 | 7 |
| Carlsson,A.C.(2013) | 0 | 1 | 0 | 1 | 1 | 1 | 1 | 1 | 0 | 6 |
| Ahmed, H. M.(2013) | 1 | 1 | 1 | 1 | 1 | 1 | 1 | 0 | 1 | 8 |
| Hoevenaar-Blom, M. P.(2013) | 1 | 1 | 1 | 1 | 1 | 1 | 1 | 1 | 0 | 8 |
| Wang, Y.(2011) | 1 | 1 | 0 | 1 | 1 | 1 | 1 | 1 | 1 | 8 |
| Zhang, Y.(2011) | 1 | 1 | 0 | 1 | 1 | 1 | 1 | 1 | 1 | 8 |
| Ford, E. S.(2009) | 1 | 1 | 0 | 1 | 1 | 1 | 1 | 0 | 1 | 7 |
| Cardi, M.(2009) | 0 | 1 | 0 | 1 | 1 | 1 | 1 | 1 | 0 | 6 |
| Lee, C. D.(2009) | 0 | 1 | 1 | 0 | 1 | 1 | 1 | 1 | 0 | 6 |
| Myint, P. K.(2009) | 0 | 1 | 1 | 1 | 1 | 1 | 1 | 1 | 0 | 7 |
| Kurth, T.(2006) | 0 | 1 | 1 | 1 | 1 | 1 | 1 | 1 | 1 | 8 |
| Troeschel, A. N.(2023) | 1 | 1 | 1 | 0 | 1 | 1 | 1 | 1 | 1 | 8 |
| Wang, T.(2022) | 1 | 1 | 0 | 0 | 1 | 1 | 1 | 0 | 0 | 5 |
| Heath, L.(2022) | 1 | 1 | 0 | 1 | 1 | 1 | 1 | 1 | 0 | 7 |
| Hu, P.(2022) | 1 | 1 | 1 | 1 | 1 | 1 | 1 | 0 | 0 | 7 |
| Kim, S.(2022) | 0 | 1 | 0 | 0 | 1 | 1 | 1 | 0 | 0 | 4 |
| Ibsen, D. B.(2021) | 1 | 1 | 1 | 0 | 1 | 1 | 1 | 1 | 0 | 7 |
| Li, Z.(2021) | 0 | 1 | 0 | 0 | 1 | 1 | 1 | 1 | 0 | 5 |
| Liu, G.(2021) | 0 | 1 | 1 | 0 | 1 | 1 | 1 | 1 | 1 | 7 |
| Sotos-Prieto, M.(2021) | 1 | 1 | 1 | 1 | 1 | 1 | 1 | 0 | 0 | 7 |
| Troeschel, A. N.(2021) | 1 | 1 | 1 | 0 | 1 | 1 | 1 | 1 | 1 | 8 |
| Zhang, X.(2021) | 1 | 1 | 1 | 1 | 1 | 1 | 1 | 0 | 0 | 7 |
| Lee, D. H.(2020) | 1 | 1 | 1 | 1 | 1 | 1 | 1 | 0 | 0 | 7 |
| Wu, M. Y.(2020) | 1 | 1 | 1 | 0 | 1 | 1 | 1 | 1 | 0 | 7 |
| Bonaccio, M.(2019) | 1 | 1 | 1 | 0 | 1 | 1 | 1 | 0 | 1 | 7 |
| Zhu, N.(2019) | 1 | 1 | 1 | 0 | 1 | 1 | 1 | 1 | 0 | 7 |
| Han, C.(2018) | 1 | 1 | 1 | 1 | 1 | 1 | 1 | 0 | 1 | 8 |
| Li, Y.(2018) | 1 | 1 | 1 | 0 | 1 | 1 | 1 | 1 | 1 | 8 |
| Zhang, Q. L.(2017) | 0 | 1 | 1 | 0 | 1 | 1 | 1 | 0 | 1 | 6 |
| Fazel-Tabar Malekshah, A.(2016) | 1 | 1 | 1 | 1 | 1 | 1 | 1 | 0 | 1 | 8 |
| Lohse, T.(2016) | 1 | 1 | 0 | 0 | 1 | 1 | 1 | 1 | 0 | 6 |
| Warren Andersen, S.(2016 AF) | 1 | 1 | 0 | 0 | 1 | 1 | 1 | 0 | 0 | 5 |
| Warren Andersen, S.(2016 WH) | 1 | 1 | 0 | 0 | 1 | 1 | 1 | 0 | 0 | 5 |
| Taheri, Shahrad(2015 EU) | 1 | 1 | 0 | 1 | 1 | 1 | 1 | 1 | 1 | 8 |
| Taheri, Shahrad(2015 SA) | 1 | 1 | 0 | 1 | 1 | 1 | 1 | 1 | 1 | 8 |
| Hoevenaar-Blom, M. P.(2014) | 1 | 1 | 0 | 0 | 1 | 1 | 1 | 1 | 0 | 6 |
| Eguchi, E.(2012) | 1 | 1 | 1 | 1 | 1 | 1 | 1 | 1 | 0 | 8 |
| Ford, E. S.(2011) | 0 | 1 | 1 | 0 | 1 | 1 | 1 | 1 | 0 | 6 |
| McCullough, M. L.(2011) | 0 | 1 | 1 | 0 | 1 | 1 | 1 | 1 | 0 | 6 |
| Odegaard, A. O.(2011) | 1 | 1 | 1 | 1 | 1 | 1 | 1 | 1 | 0 | 8 |
| Kvaavik, E.(2010) | 1 | 1 | 1 | 1 | 1 | 1 | 1 | 1 | 1 | 9 |
| Mitchell, J. A.(2010) | 0 | 1 | 1 | 0 | 1 | 1 | 1 | 1 | 0 | 6 |
| Nechuta, S. J.(2010) | 0 | 1 | 1 | 0 | 1 | 1 | 1 | 0 | 1 | 6 |
| Lee, C. D.(2009) | 0 | 1 | 1 | 1 | 1 | 1 | 1 | 1 | 0 | 7 |
| Khaw, K. T.(2008) | 0 | 1 | 1 | 1 | 1 | 1 | 1 | 1 | 0 | 7 |
| Knoops, K. T.(2004) | 0 | 1 | 1 | 1 | 1 | 1 | 1 | 1 | 1 | 8 |
| Luoto, R.(1998) | 1 | 1 | 0 | 0 | 1 | 1 | 1 | 1 | 1 | 7 |
| Yang, Y. L.(2021) | 1 | 1 | 1 | 1 | 1 | 1 | 1 | 1 | 0 | 8 |
| Booth, J. N.(2014) | 1 | 1 | 1 | 1 | 1 | 1 | 1 | 0 | 0 | 7 |
| Towfighi, A.(2012) | 1 | 1 | 1 | 1 | 1 | 1 | 1 | 0 | 0 | 7 |
| Han, Y.(2021) | 1 | 1 | 1 | 1 | 1 | 1 | 1 | 1 | 0 | 8 |
| Booth, J. N.(2014) | 1 | 1 | 1 | 1 | 1 | 1 | 1 | 0 | 0 | 7 |
| Towfighi, A.(2012) | 1 | 1 | 1 | 1 | 1 | 1 | 1 | 0 | 0 | 7 |
| Term1. Representativeness of the exposed cohort. | | | | | | | | | | |
| Term2. Selection of the non-exposed cohort. | | | | | | | | | | |
| Term3. Ascertainment of exposure. | | | | | | | | | | |
| Term4. Demonstration that outcome of interest was not present at start of study. | | | | | | | | | | |
| Term5. Comparability of cohorts on the basis of the design or analysis (adjusted for age). | | | | | | | | | | |
| Term6. Comparability of cohorts on the basis of the design or analysis (adjusted for other confounding factor). | | | | | | | | | | |
| Term7. Assessment of outcome. | | | | | | | | | | |
| Term8. Was follow-up long enough for outcomes to occur. | | | | | | | | | | |
| Term9. Adequacy of follow-up of cohort. | | | | | | | | | | |

**Supplemental Table 6.** Subgroup analyses of LBs and risk of CVD.

| **Subgroups** | **Studies** | **Participants** | **Cases** | **RR (95% CI)** | ***I^2^*** | ***P_heter_*** | ***P_reg_*** |
| --- | --- | --- | --- | --- | --- | --- | --- |
| Sub-types of outcomes | | | |  | | | 0.736 |
| CVD | 11 | 51,521 | 606,707 | 0.45(0.37-0.55) | 93.20% | <0.01 |  |
| CHD | 8 | 16,620 | 718,201 | 0.34(0.22-0.52) | 90.60% | <0.01 |  |
| Stroke | 11 | 57,993 | 1,171,896 | 0.50(0.42-0.61) | 88.30% | <0.01 |  |
| MI | 3 | 3,094 | 138,705 | 0.36(0.10-1.27) | 87.30% | <0.01 |  |
| HF | 6 | 11,081 | 678,978 | 0.37(0.24-0.56) | 92.90% | <0.01 |  |
| IHD | 1 | 34,304 | 461,047 | 0.50(0.45-0.54) | 0% | - |  |
| Continent | | | |  | | | 0.387 |
| America | 10 | 18,474 | 358,283 | 0.33(0.20-0.52) | 92.60% | <0.01 |  |
| Europe | 14 | 43,374 | 654,423 | 0.41(0.33-0.50) | 88.70% | <0.01 |  |
| Asia | 4 | 127,885 | 1,510,328 | 0.61(0.47-0.80) | 95.40% | <0.01 |  |
| Sex | | | |  | | | 0.048 |
| Both | 18 | 165,893 | 2,048,471 | 0.53(0.47-0.61) | 89.60% | <0.01 |  |
| Men | 5 | 11,474 | 133,791 | 0.33(0.20-0.57) | 92.40% | <0.01 |  |
| Women | 6 | 12,366 | 340,772 | 0.25(0.16-0.39) | 78.90% | <0.01 |  |
| Follow-up years | | | |  | | | 0.544 |
| <10 | 3 | 45,303 | 490,593 | 0.49(0.26-0.91) | 49.40% | 0.139 |  |
| ≥10 | 26 | 144,430 | 2,032,441 | 0.42(0.37-0.48) | 93.20% | <0.01 |  |
| Average age | | | |  | | | 0.524 |
| ≥60 | 8 | 8,401 | 177,836 | 0.45(0.30-0.66) | 88.70% | <0.01 |  |
| <60 | 21 | 181,332 | 2,345,198 | 0.42(0.36-0.49) | 93.00% | <0.01 |  |
| Types of LBs included in the original study | | | | | | | |
| Smoking |  |  |  |  |  |  | - |
| Yes | 29 | 189733 | 2,523,034 | 0.42(0.37-0.48) | 92.50% | <0.001 |  |
| No | - |  |  | - | - | - |  |
| Alcohol drinking | | | | | | | 0.718 |
| Yes | 21 | 179574 | 2,256,371 | 0.45(0.39-0.53) | 92.20% | <0.001 |  |
| No | 8 | 10159 | 266,663 | 0.38(0.27-0.53) | 88.60% | <0.001 |  |
| PA |  |  |  |  |  |  | - |
| Yes | 29 | 189733 | 2,523,034 | 0.42(0.37-0.48) | 92.50% | <0.001 |  |
| No | - |  |  | - | - | - |  |
| Diet |  |  |  |  | | | 0.919 |
| Yes | 28 | 189484 | 2,522,071 | 0.42(0.37-0.48) | 92.70% | <0.001 |  |
| No | 1 | 249 | 963 | 0.36(0.07-1.93) | 0.00% | - |  |
| Body weight |  |  |  |  | | | 0.187 |
| Yes | 24 | 152017 | 2,049,870 | 0.38(0.32-0.46) | 88.10% | <0.001 |  |
| No | 5 | 37716 | 473,164 | 0.61(0.52-0.72) | 89.90% | <0.001 |  |
| Adjustment | | | |  |  |  |  |
| Age |  |  |  |  | | | 0.556 |
| Yes | 25 | 184492 | 2,007,980 | 0.41(0.36-0.48) | 93.30% | <0.001 |  |
| No | 4 | 5241 | 515,054 | 0.49(0.33-0.73) | 71.60% | 0.014 |  |
| Economic level |  |  |  |  | | | 0.012 |
| Yes | 6 | 10861 | 164,695 | 0.70(0.59-0.83) | 57.10% | 0.040 |  |
| No | 23 | 178872 | 2,358,339 | 0.37(0.31-0.43) | 92.90% | <0.01 |  |
| Educational level |  |  |  |  | | | 0.013 |
| Yes | 21 | 175739 | 2,267,505 | 0.48(0.43-0.55) | 91.90% | <0.01 |  |
| No | 8 | 13994 | 255,529 | 0.25(0.15-0.43) | 81.00% | <0.01 |  |

Abbreviations: CI, confidence interval; CVD, cardiovascular disease; CHD, coronary heart disease; HF, heart failure; IHD, ischemic heart disease; LB, lifestyle behavior; MI, myocardial infarction; PA, physical activity; RR, relative risk.

*P*_heter_, *P* for heterogeneity within each subgroup.

*P*_reg_, *P* for heterogeneity between subgroups using meta-regression analyses.

**Supplemental Table 7.** Subgroup analyses of per 1 healthy LB increment and risk of CVD.

| Subgroups | Studies | Participants | Cases | RR (95% CI) | *I^2^* | *P_heter_* | *P_reg_* |
| --- | --- | --- | --- | --- | --- | --- | --- |
| Sub-types of outcomes |  |  |  |  | | | 0.846 |
| CVD | 9 | 49,736 | 575,829 | 0.83(0.82-0.89) | 95.90% | <0.001 |  |
| CHD | 4 | 7,588 | 517,430 | 0.82(0.79-0.86) | 47.40% | 0.127 |  |
| Stroke | 4 | 41,358 | 549,469 | 0.85(0.81-0.88) | 79.00% | 0.003 |  |
| MI | - | - | - | - |  |  |  |
| HF | 6 | 49,736 | 575,829 | 0.77(0.70-0.85) | 95.60% | <0.001 |  |
| IHD | 1 | 34,304 | 461,047 | 0.85(0.84-0.87) | 0% | - |  |
| Continent |  |  |  |  | | | 0.178 |
| America | 7 | 16,005 | 216,240 | 0.82(0.78-0.86) | 86.50% | <0.001 |  |
| Europe | 11 | 41,382 | 595,138 | 0.81(0.76-0.86) | 95.80% | <0.001 |  |
| Asia | 5 | 86,680 | 1,510,328 | 0.87(0.82-0.91) | 96.10% | <0.001 |  |
| Sex |  |  |  |  | | | 0.209 |
| Both | 15 | 122,494 | 1,994,440 | 0.84(0.82-0.87) | 95.50% | <0.001 |  |
| Men | 4 | 10,113 | 113,070 | 0.81(0.79-0.83) | 35.60% | 0.199 |  |
| Women | 4 | 11,460 | 214,196 | 0.79(0.71-0.86) | 93.90% | <0.001 |  |
| Follow-up years |  |  |  |  | | | 0.672 |
| <10 | 2 | 3,689 | 467,440 | 0.84(0.76-0.94) | 64.30% | 0.094 |  |
| ≥10 | 21 | 140,378 | 1,854,266 | 0.82(0.80-0.85) | 96.40% | <0.001 |  |
| Average age |  |  |  |  | | | 0.754 |
| ≥60 | 7 | 6,838 | 162,369 | 0.83(0.77-0.90) | 91.30% | <0.001 |  |
| <60 | 16 | 137,229 | 2,159,337 | 0.82(0.80-0.85) | 96.70% | <0.001 |  |
| Types of LBs included in the original study | | | | | | | |
| Smoking |  |  |  |  | | | - |
| Yes | 23 | 144,067 | 2,321,706 | 0.83(0.80-0.85) | 96.20% | <0.001 |  |
| No | 0 |  |  | - | - | - |  |
| Alcohol dringking |  |  |  |  | | | 0.005 |
| Yes | 16 | 134,317 | 2,078,196 | 0.85(0.83-0.88) | 95.70% | <0.001 |  |
| No | 7 | 9,750 | 243,510 | 0.77(0.72-0.82) | 88.10% | <0.001 |  |
| PA |  |  |  |  |  |  | - |
| Yes | 23 | 144,067 | 2,321,706 | 0.83(0.80-0.85) | 96.20% | <0.001 |  |
| No | 0 |  |  | - | - | - |  |
| Diet |  |  |  |  | | | 0.183 |
| Yes | 22 | 143,818 | 2,320,743 | 0.82(0.80-0.85) | 96.40% | <0.001 |  |
| No | 1 | 249 | 963 | 0.94(0.84-1.05) | 0.00% | - |  |
| Body weight |  |  |  |  | | | 0.214 |
| Yes | 18 | 106,351 | 1,848,542 | 0.81(0.79-0.84) | 89.90% | <0.001 |  |
| No | 5 | 37,716 | 473,164 | 0.87(0.84-0.91) | 92.30% | <0.001 |  |
| Adjustment |  |  |  |  |  |  |  |
| Age |  |  |  |  | | | 0.099 |
| Yes | 20 | 139,235 | 1,829,805 | 0.82(0.79-0.84) | 96.70% | <0.001 |  |
| No | 3 | 4,832 | 491,901 | 0.89(0.86-0.91) | 16.90% | 0.300 |  |
| Economic level |  |  |  |  | | | 0.112 |
| Yes | 4 | 8,848 | 111,592 | 0.88(0.82-0.95) | 82.70% | 0.001 |  |
| No | 19 | 135,219 | 2,210,114 | 0.81(0.79-0.84) | 96.70% | <0.001 |  |
| Educational level |  |  |  |  | | | 0.929 |
| Yes | 16 | 130,529 | 2,155,117 | 0.83(0.80-0.85) | 96.70% | <0.001 |  |
| No | 7 | 13,538 | 166,589 | 0.82(0.79-0.85) | 49.90% | 0.063 |  |

Abbreviations: CI, confidence interval; CVD, cardiovascular disease; CHD, coronary heart disease; HF, heart failure; IHD, ischemic heart disease; LB, lifestyle behavior; MI, myocardial infarction; PA, physical activity; RR, relative risk.

*P*_heter_, *P* for heterogeneity within each subgroup.

*P*_reg_, *P* for heterogeneity between subgroups using meta-regression analyses.

**Supplemental Table 8.** Subgroup analyses of LBs and risk of CVD mortality.

| Subgroups | Studies | Participants | Cases | RR (95% CI) | *I^2^* | *P_heter_* | *P_reg_* |
| --- | --- | --- | --- | --- | --- | --- | --- |
| Sub-types of outcomes |  |  |  |  | | | 0.355 |
| CVD | 33 | 56,643 | 2,635,115 | 0.43(0.38-0.49) | 87.10% | <0.001 |  |
| CHD | 5 | 2,058 | 91,440 | 0.26(0.19-0.34) | 0.00% | 0.632 |  |
| Stroke | 2 |  |  | 0.33(0.24-0.43) | 0.00% | 0.909 |  |
| MI | - | - | - | - | - | - |  |
| HF | - | - | - | - | - | - |  |
| IHD | 1 | 5,116 | 487,198 | 0.42(0.26-0.67) | 0.00% | - |  |
| Continent |  |  |  |  | | | 0.221 |
| America | 10 | 29,763 | 480,378 | 0.42(0.34-0.51) | 89.00% | <0.001 |  |
| Europe | 11 | 10,609 | 660,651 | 0.41(0.31-0.54) | 88.40% | <0.001 |  |
| Asia | 15 | 27,839 | 2,135,625 | 0.51(0.41-0.63) | 92.30% | <0.001 |  |
| Sex |  |  |  |  | | | 0.132 |
| Both | 30 | 58,003 | 3,047,708 | 0.47(0.40-0.54) | 94.00% | <0.001 |  |
| Men | 3 | 2,892 | 121,514 | 0.30(0.25-0.36) | 0.40% | 0.367 |  |
| Women | 3 | 7,316 | 107,432 | 0.50(0.36-0.70) | 56.20% | 0.102 |  |
| Follow-up years |  |  |  |  | | | 0.392 |
| <10 | 13 | 11,862 | 1,424,463 | 0.49(0.40-0.62) | 72.10% | <0.001 |  |
| ≥10 | 23 | 56,349 | 1,852,191 | 0.43(0.37-0.51) | 96.00% | <0.001 |  |
| Average age |  |  |  |  | | | 0.144 |
| ≥60 | 7 | 16,106 | 142,496 | 0.55(0.47-0.65) | 82.60% | <0.001 |  |
| <60 | 29 | 52,105 | 3,134,158 | 0.42(0.35-0.50) | 94.20% | <0.001 |  |
| Types of LBs included in the original study | | | | | | | |
| Smoking |  |  |  |  | | | 0.136 |
| Yes | 33 | 60,348 | 3,170,117 | 0.43(0.37-0.50) | 94.30% | <0.001 |  |
| No | 3 | 7,863 | 106,537 | 0.63(0.38-1.05) | 95.90% | <0.001 |  |
| Alcohol dringking |  |  |  |  | | | 0.905 |
| Yes | 30 | 63,061 | 3,005,246 | 0.45(0.39-0.52) | 94.90% | <0.001 |  |
| No | 6 | 5,150 | 271,408 | 0.45(0.30-0.68) | 86.70% | <0.001 |  |
| PA |  |  |  |  |  |  | - |
| Yes | 36 |  |  | 0.45(0.39-0.51) | 94.40% | <0.001 |  |
| No | - | - |  | - | - | - |  |
| Diet |  |  |  |  | | | 0.217 |
| Yes | 28 | 53,930 | 2,656,740 | 0.42(0.35-0.50) | <0.001 | <0.001 |  |
| No | 8 | 14,281 | 619,914 | 0.56(0.47-0.66) | 76.70% | <0.001 |  |
| Body weight |  |  |  |  | | | 0.282 |
| Yes | 20 | 48,893 | 1,498,661 | 0.48(0.41-0.58) | <0.001 | <0.001 |  |
| No | 16 | 19,318 | 1,777,993 | 0.42(0.36-0.49) | 75.00% | <0.001 |  |
| Adjustment |  |  |  |  |  |  |  |
| Age |  |  |  |  | | | 0.382 |
| Yes | 32 | 54,137 | 2,618,095 | 0.44(0.38-0.51) | 94.70% | <0.001 |  |
| No | 4 | 14,074 | 658,559 | 0.53(0.30-0.92) | 92.40% | <0.001 |  |
| Economic level |  |  |  |  | | | 0.293 |
| Yes | 11 | 12,192 | 1,330,577 | 0.51(0.40-0.64) | 78.2% | <0.001 |  |
| No | 25 | 41,077 | 1,514,402 | 0.43(0.36-0.50) | 95.70% | <0.001 |  |
| Educational level |  |  |  |  | | | 0.015 |
| Yes | 26 | 52,673 | 3,033,452 | 0.51(0.44-0.59) | 94.60% | <0.001 |  |
| No | 10 | 15,538 | 243,202 | 0.33(0.25-0.44) | 78.00% | <0.001 |  |

Abbreviations: CI, confidence interval; CVD, cardiovascular disease; CHD, coronary heart disease; HF, heart failure; IHD, ischemic heart disease; LB, lifestyle behavior; MI, myocardial infarction; PA, physical activity; RR, relative risk.

*P*_heter_, *P* for heterogeneity within each subgroup.

*P*_reg_, *P* for heterogeneity between subgroups using meta-regression analyses.

**Supplemental Table 9.** Subgroup analyses of per 1 healthy LB increment and risk of CVD mortality.

| Subgroups | Studies | Participants | Cases | RR (95% CI) | *I^2^* | *P_heter_* | *P_reg_* |
| --- | --- | --- | --- | --- | --- | --- | --- |
| Sub-types of outcomes |  |  |  |  | | | 0.304 |
| CVD | 29 | 44,466 | 2,302,565 | 0.80(0.77-0.83) | 96.30% | <0.001 |  |
| CHD | 5 | 2,058 | 91,440 | 0.80(0.74-0.85) | 56.60% | 0.056 |  |
| Stroke | 2 | 6,930 | 530,208 | 0.82(0.78-0.86) | 70.70% | 0.065 |  |
| MI | - |  |  | - |  |  |  |
| HF | - |  |  | - |  |  |  |
| IHD | 1 | 5,116 | 487,198 | 0.77(0.75-0.80) | 0.00% | - |  |
| Continent |  |  |  |  | | | 0.034 |
| America | 7 | 20,803 | 411,274 | 0.78(0.70-0.86) | 98.80% | <0.001 |  |
| Europe | 10 | 10,535 | 649,561 | 0.77(0.70-0.85) | 95.00% | <0.001 |  |
| Asia | 14 | 24,696 | 1,804,168 | 0.84(0.81-0.88) | 83.50% | <0.001 |  |
| Sex |  |  |  |  | | | 0.143 |
| Both | 26 | 52,400 | 2,669,212 | 0.81(0.78-0.85) | 96.70% | <0.001 |  |
| Men | 3 | 2,892 | 121,514 | 0.73(0.67-0.80) | 83.60% | 0.002 |  |
| Women | 2 | 742 | 74,277 | 0.85(0.75-0.95) | 72.80% | 0.055 |  |
| Follow-up years |  |  |  |  | | | 0.097 |
| <10 | 12 | 11,788 | 1,413,373 | 0.84(0.80-0.88) | 79.00% | <0.001 |  |
| ≥10 | 19 | 44,246 | 1,451,630 | 0.79(0.75-0.82) | 97.60% | <0.001 |  |
| Average age |  |  |  |  | | | 0.193 |
| ≥60 | 4 | 7,146 | 73,392 | 0.86(0.80-0.93) | 82.50% | 0.001 |  |
| <60 | 27 | 48,888 | 2,791,611 | 0.80(0.77-0.83) | 95.20% | <0.001 |  |
| Types of LBs included in the original study | | | | | | | |
| Smoking |  |  |  |  | | | 0.040 |
| Yes | 28 | 48,171 | 2758466 | 0.80(0.77-0.83) | 95.00% | <0.001 |  |
| No | 3 | 7,863 | 106537 | 0.90(0.85-0.96) | 69.70% | 0.037 |  |
| Alcohol dringking |  |  |  |  | | | 0.795 |
| Yes | 25 | 50,884 | 2593595 | 0.81(0.78-0.84) | 96.80% | <0.001 |  |
| No | 6 | 5,150 | 271408 | 0.80(0.72-0.88) | 89.40% | <0.001 |  |
| PA |  |  |  |  |  |  | - |
| Yes | 31 | 56,034 | 2865003 | 0.81(0.78-0.84) | 96.20% | <0.001 |  |
| No | - |  |  | - |  |  |  |
| Diet |  |  |  |  | | | 0.648 |
| Yes | 26 | 52,686 | 2628185 | 0.80(0.77-0.84) | 96.80% | <0.001 |  |
| No | 5 | 3,348 | 236818 | 0.82(0.76-0.89) | 82.30% | <0.001 |  |
| Body weight |  |  |  |  | | | 0.209 |
| Yes | 15 | 36,716 | 1087010 | 0.83(0.78-0.87) | 97.60% | <0.001 |  |
| No | 16 | 19,318 | 1777993 | 0.79(0.74-0.83) | 92.60% | <0.001 |  |
| Adjustment |  |  |  |  |  |  |  |
| Age |  |  |  |  | | | 0.410 |
| Yes | 27 | 41,960 | 2206444 | 0.80(0.77-0.83) | 96.50% | <0.001 |  |
| No | 4 | 14,074 | 658559 | 0.84(0.76-0.93) | 93.00% | <0.001 |  |
| Economic level |  |  |  |  | | | 0.254 |
| Yes | 10 | 10,976 | 1312093 | 0.83(0.79-0.87) | 78.00% | <0.001 |  |
| No | 21 | 45,058 | 1552910 | 0.79(0.76-0.83) | 97.30% | <0.001 |  |
| Educational level |  |  |  |  | | | 0.014 |
| Yes | 22 | 41,666 | 2639266 | 0.83(0.80-0.86) | 93.50% | <0.001 |  |
| No | 9 | 14,368 | 225737 | 0.74(0.71-0.78) | 76.50% | <0.001 |  |

Abbreviations: CI, confidence interval; CVD, cardiovascular disease; CHD, coronary heart disease; HF, heart failure; IHD, ischemic heart disease; LB, lifestyle behavior; MI, myocardial infarction; PA, physical activity; RR, relative risk.

*P*_heter_, *P* for heterogeneity within each subgroup.

*P*_reg_, *P* for heterogeneity between subgroups using meta-regression analys

**
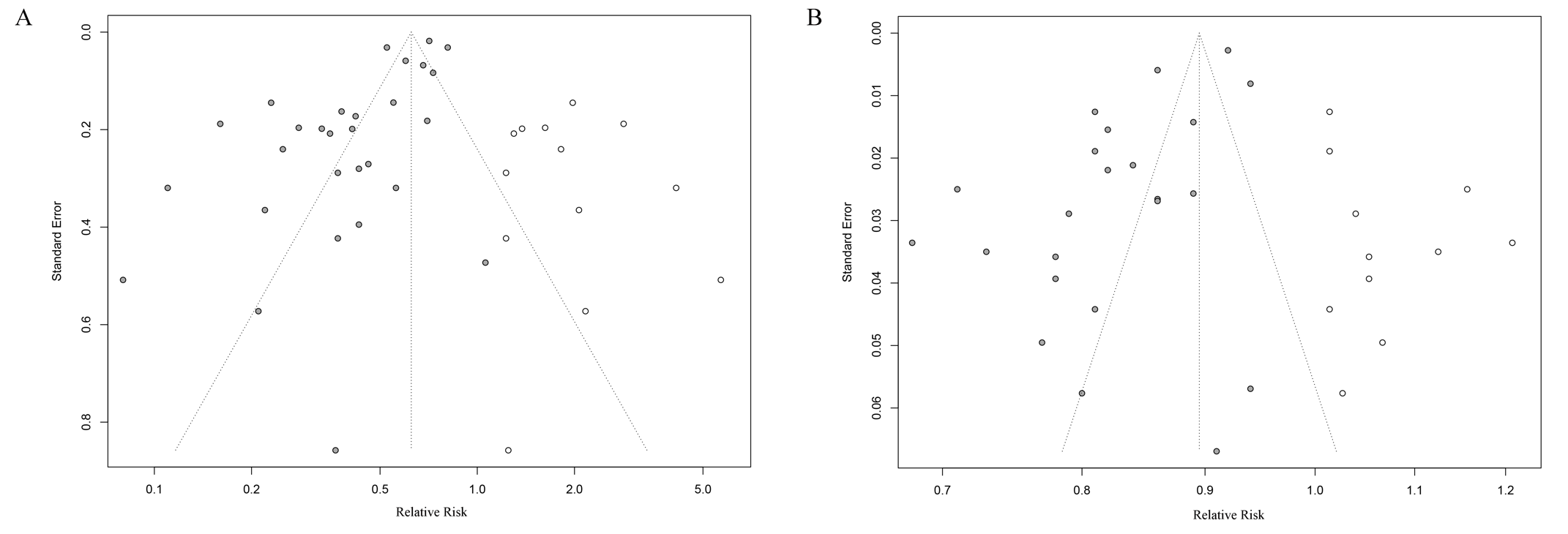
**

**Supplemental Figure 1.** Funnel plots for assessing the association between LBs and CVD (after the trim and fill analysis; A, the healthiest versus the least-healthy combination of LBs; B, per 1 healthy LB increment).

**
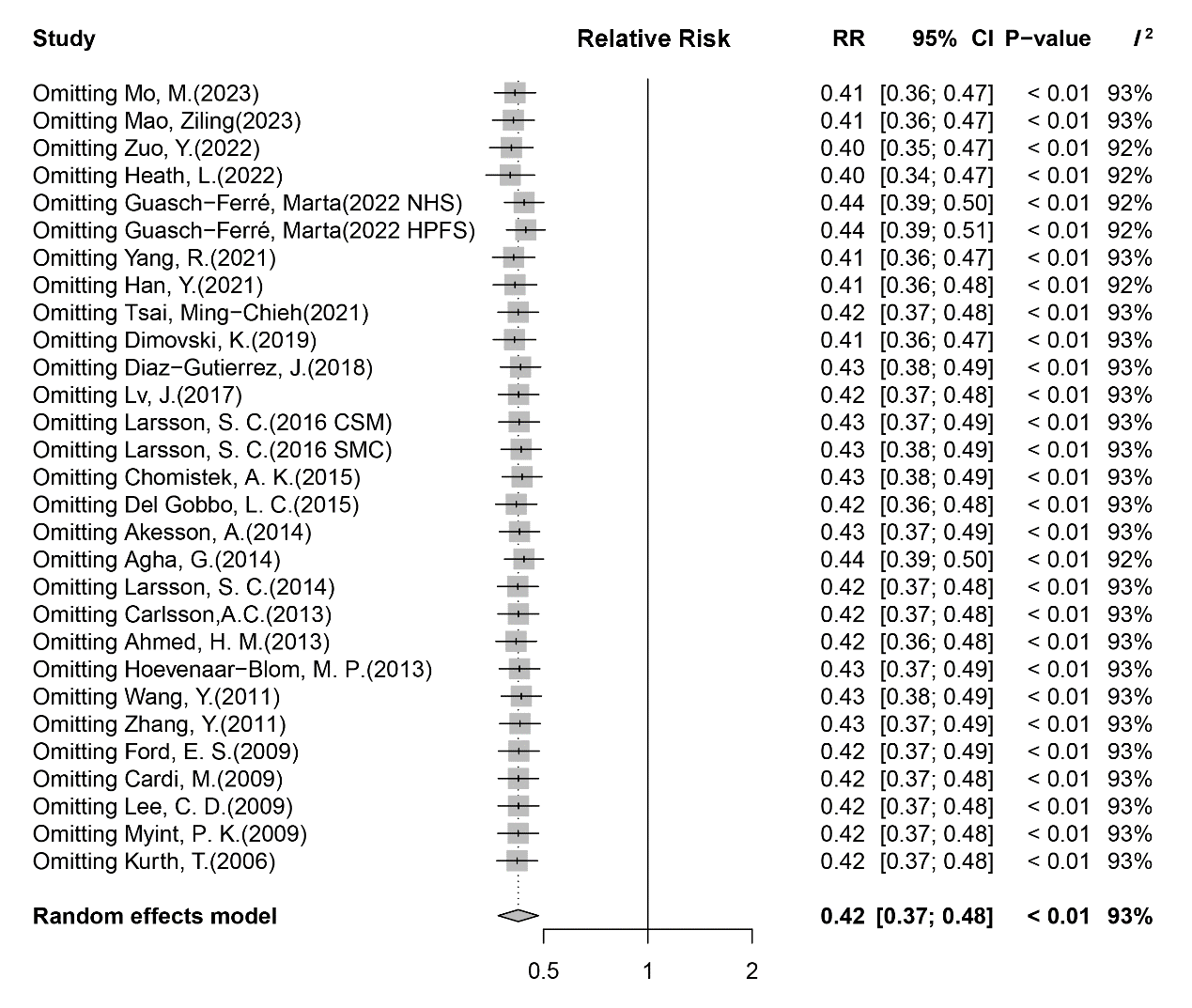
**

**Supplemental Figure 2.** Sensitivity analyses estimates (RR with corresponding 95% CI) for the associations between LBs and CVD.


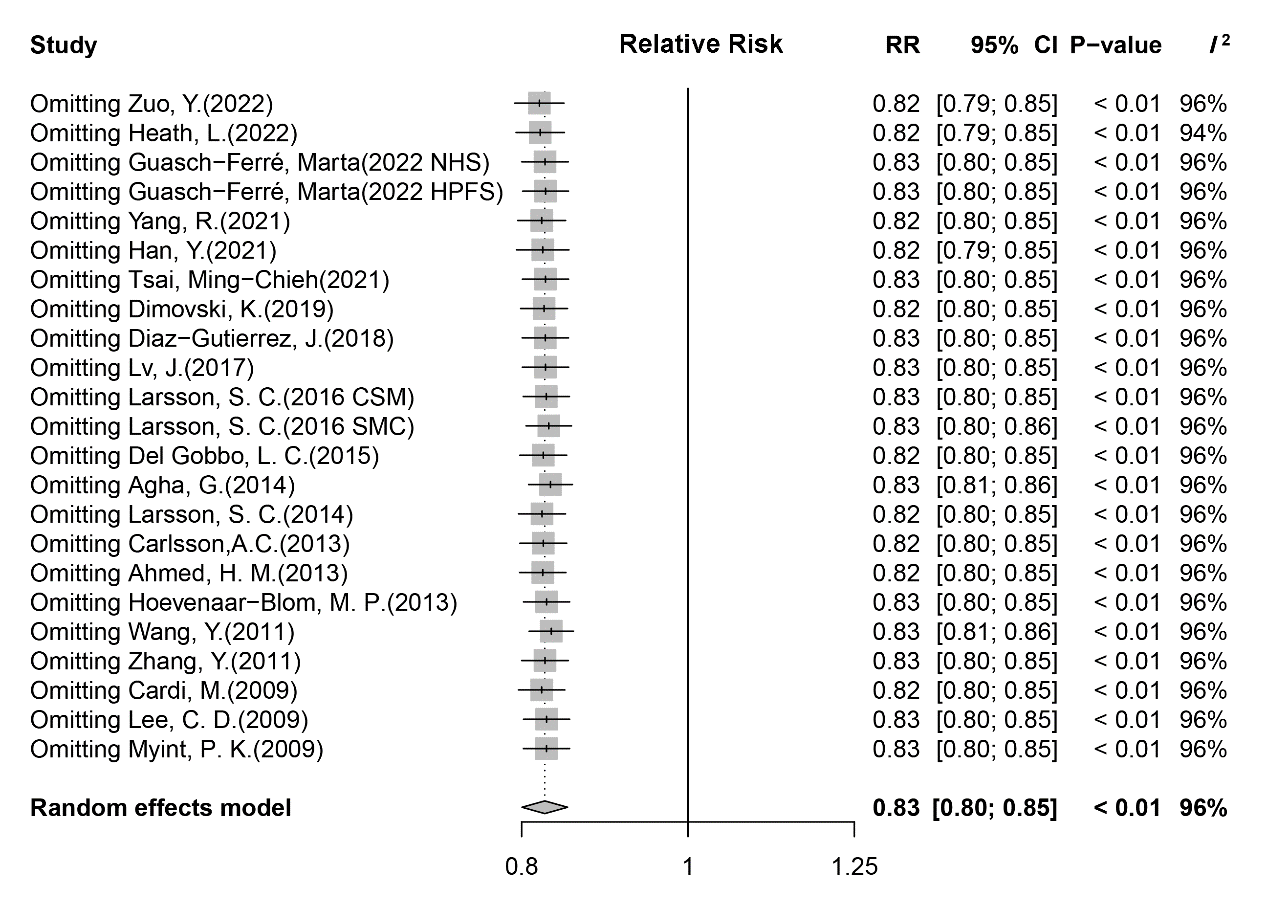


**Supplemental Figure 3.** Sensitivity analyses estimates (RR with corresponding 95% CI) for the associations between per 1 healthy LB increment and CVD.


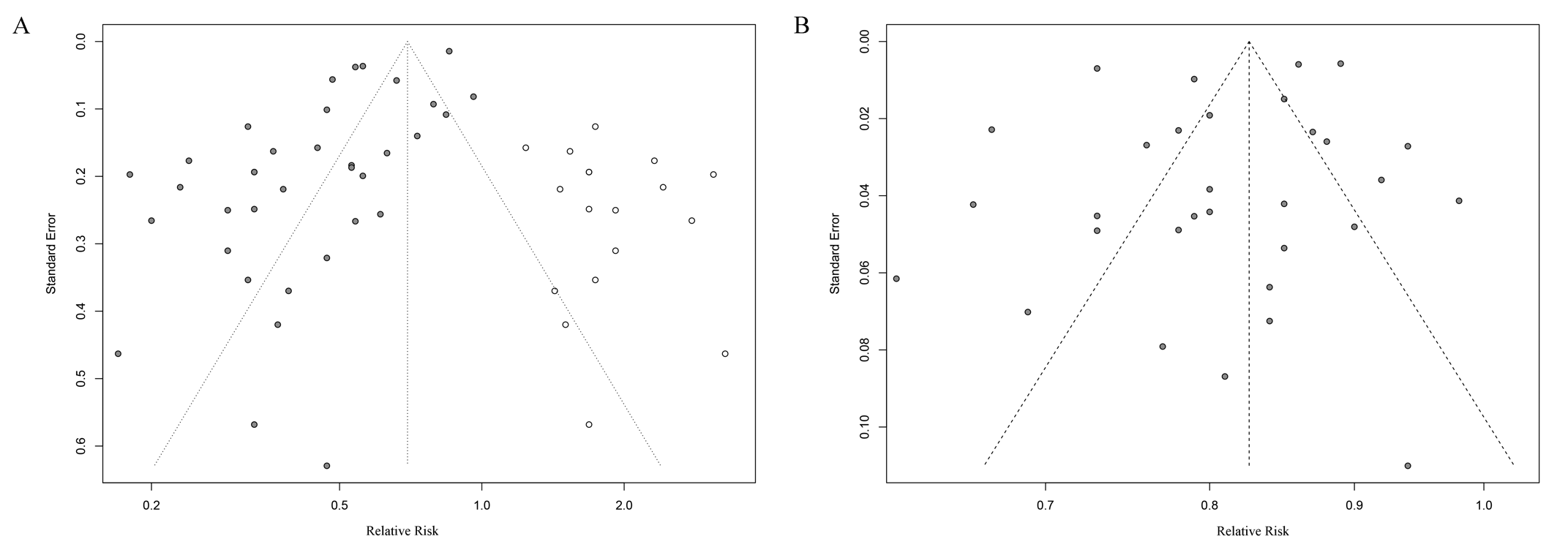


**Supplemental Figure 4.** Funnel plots for assessing the association between LBs and CVD mortality (A, the healthiest versus the least-healthy combination of LBs, after the trim and fill analysis; B, per 1 healthy LB increment).


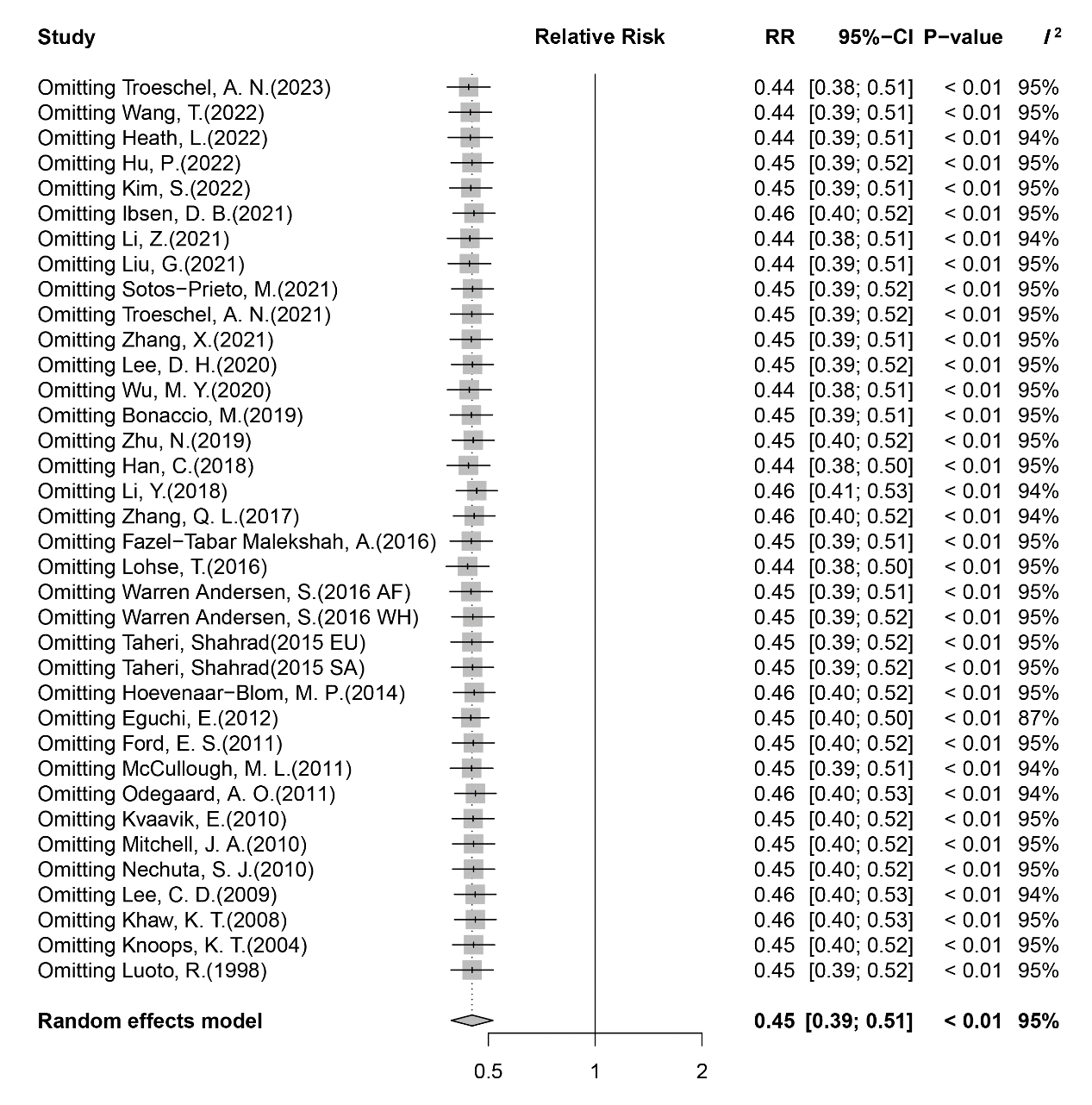


**Supplemental Figure 5.** Sensitivity analyses estimates (RR with corresponding 95% CI) for the associations between LBs and CVD mortality.


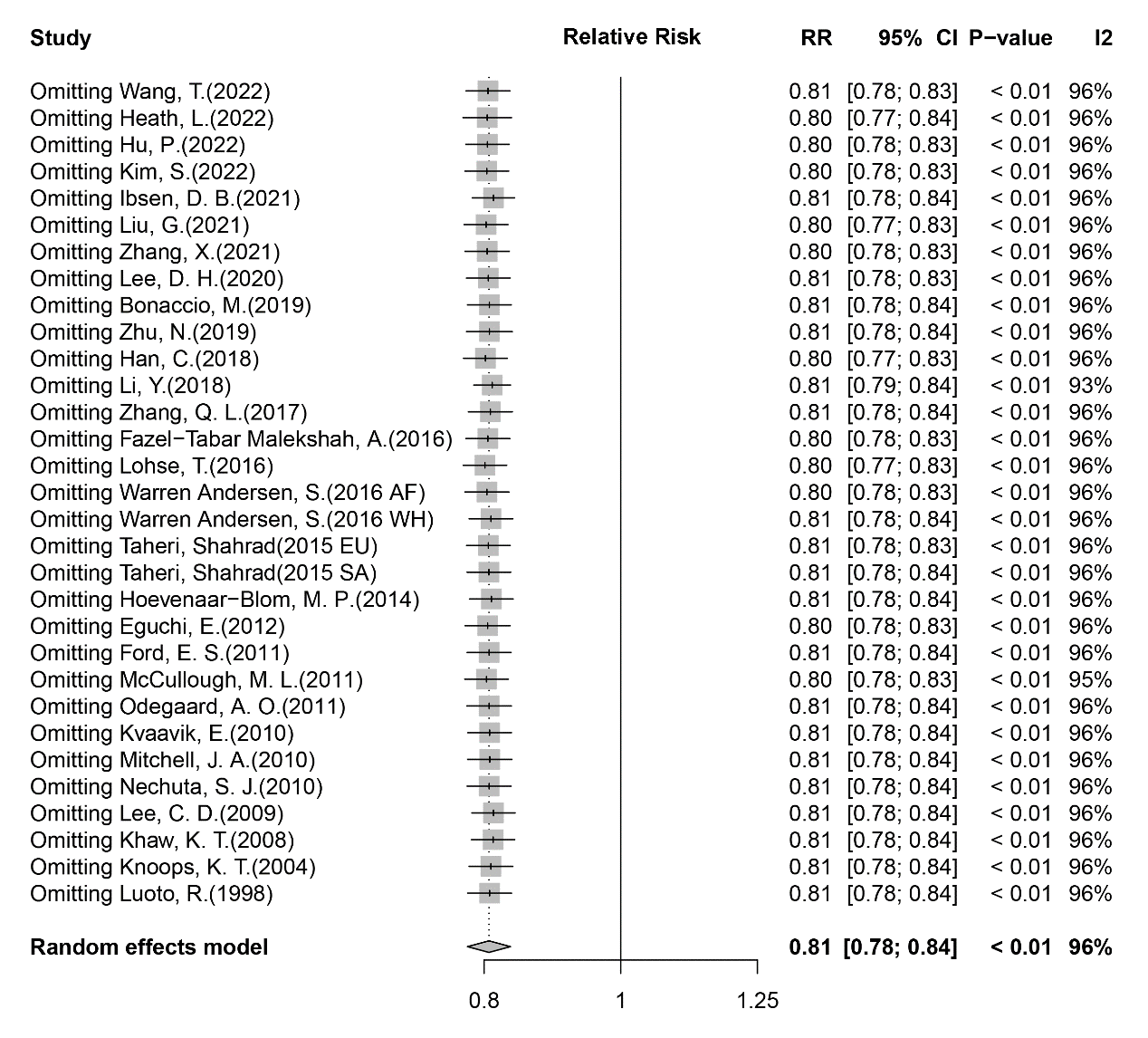


**Supplemental Figure 6.** Sensitivity analyses estimates (RR with corresponding 95% CI) for the associations between per 1 healthy LB increment and CVD mortality.


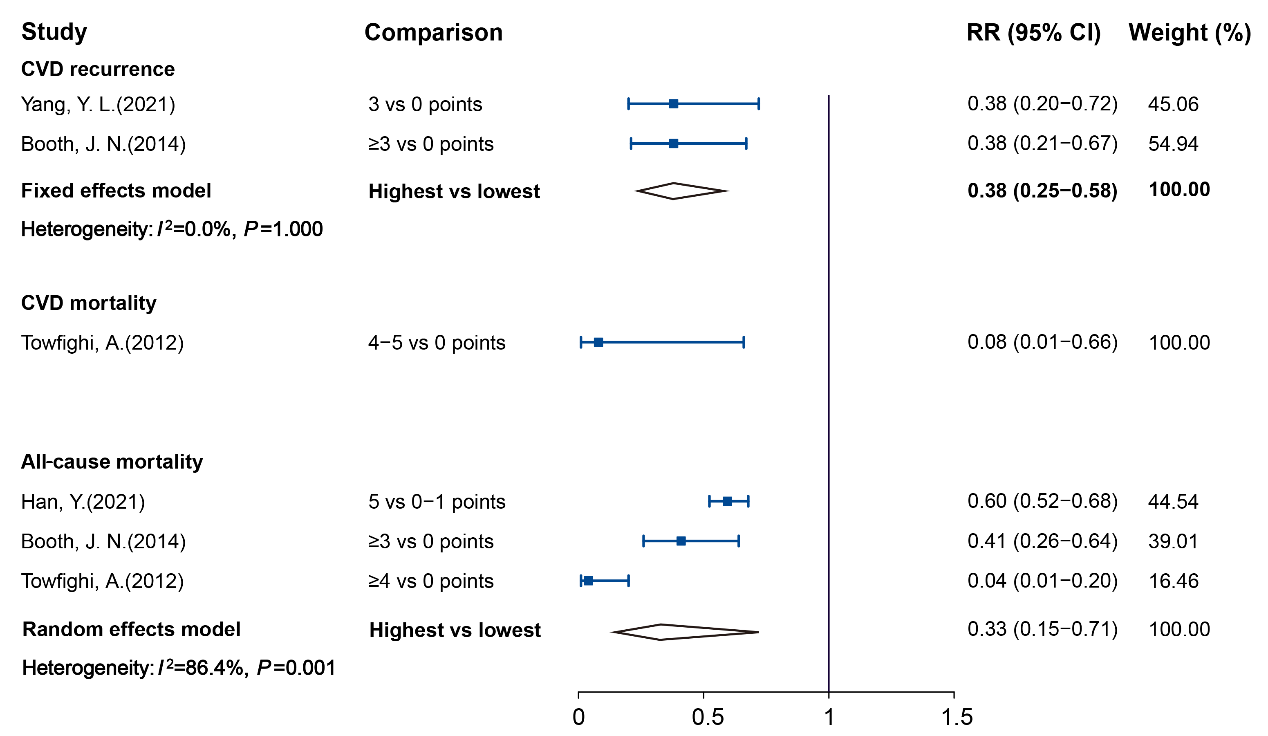


**Supplemental Figure 7.** Forest plot of pooled relative risk for CVD recurrence, mortality and all-cause mortality among individuals with CVD with the healthiest versus the least-healthy combination of LBs.


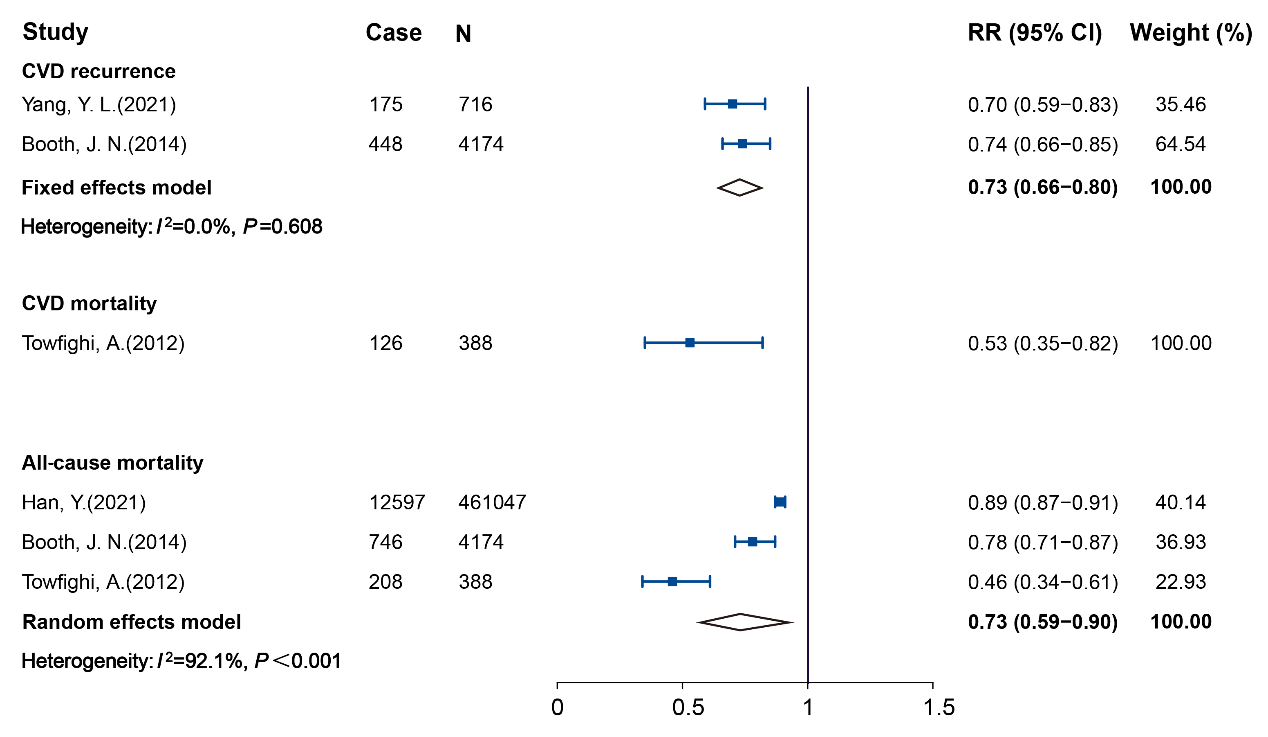


**Supplemental Figure 8.** Forest plot for the pooled associations between per 1 healthy LB increment and CVD recurrence, mortality and all-cause mortality among individuals with CVD.
