# Supplementary material for: Lifestyle behaviors and risk of cardiovascular disease and prognosis among individuals with cardiovascular disease: a systematic review and meta-analysis of 71 prospective cohort studies
Source: Int J Behav Nutr Phys Act. 2024 Apr 22;21:42. doi: 10.1186/s12966-024-01586-7 (PMC11036700; doi:10.1186/s12966-024-01586-7)
Supplement: Supplementary file 2 — Supplementary Material 2 [file 12966_2024_1586_MOESM2_ESM.doc]

March 18, 2024

RE: IJBN-D-23-00935R1-Lifestyle behaviors and risk of cardiovascular disease and prognosis among individuals with cardiovascular disease: a systematic review and meta-analysis of 71 prospective cohort studies”

Dear Editor and Reviewer,

Thanks for your and the reviewers’ careful consideration of our manuscript. We appreciate your and reviewers’ valuable and constructive comments of our manuscript. We have carefully considered them, and our point-by-point response to these comments is herein enclosed.

All authors have read and approved the final revised version of the manuscript. We hope that our responses to your comments have satisfactorily addressed your concerns and that our paper can be accepted for publication in ***International Journal of Behavioral Nutrition and Physical Activity***. We look forward to hearing from you!

Yours sincerely,

Yudong Miao, MD, PHD

Department of Health management, College of Public Health, Zhengzhou University, 100 Kexue Road, Gaoxin district, Zhengzhou, Henan, 450001, People’s Republic of China

**Telephone:** +8613939026699

**E-mail:** meldon@zzu.edu.cn

**NOTE:**

**PAGE NUMBERS REFER TO THE REVISED MANUSCRIPT WITH CHANGES MARKED.**

**Changes are highlighted in yellow**

**Associate Editor** ________________________________________________________________________

**Point 1:** *Are you from the Republic of China, 中華民國? If not, please correct your affiliation.*

**Response:** We apologize for the misunderstanding. We are from the People’s Republic of China. All affiliations of authors from the People’s Republic of China have been corrected in revised manuscript (Pages 1-2, lines 18-35).

________________________________________________________________________

**Reviewer #1** ________________________________________________________________________

**Point 1:**	*This reviewer has no further comment.*

**Response:** Thank you for your recognition of our work.

________________________________________________________________________
